# Modeling T Cell Antigen Discrimination Based on Feedback Control of Digital ERK Responses

**DOI:** 10.1371/journal.pbio.0030356

**Published:** 2005-10-25

**Authors:** Grégoire Altan-Bonnet, Ronald N Germain

**Affiliations:** **1**Lymphocyte Biology Section, Laboratory of Immunology, National Institute of Allergy and Infectious Disease, National Institutes of Health, Bethesda, Maryland, United States of America; National Jewish Medical and Research Center/Howard Hughes Medical InstituteUnited States of America

## Abstract

T-lymphocyte activation displays a remarkable combination of speed, sensitivity, and discrimination in response to peptide–major histocompatibility complex (pMHC) ligand engagement of clonally distributed antigen receptors (T cell receptors or TCRs). Even a few foreign pMHCs on the surface of an antigen-presenting cell trigger effective signaling within seconds, whereas 1 × 10^5^–1 × 10^6^ self-pMHC ligands that may differ from the foreign stimulus by only a single amino acid fail to elicit this response. No existing model accounts for this nearly absolute distinction between closely related TCR ligands while also preserving the other canonical features of T-cell responses. Here we document the unexpected highly amplified and digital nature of extracellular signal-regulated kinase (ERK) activation in T cells. Based on this observation and evidence that competing positive- and negative-feedback loops contribute to TCR ligand discrimination, we constructed a new mathematical model of proximal TCR-dependent signaling. The model made clear that competition between a digital positive feedback based on ERK activity and an analog negative feedback involving SH2 domain-containing tyrosine phosphatase (SHP-1) was critical for defining a sharp ligand-discrimination threshold while preserving a rapid and sensitive response. Several nontrivial predictions of this model, including the notion that this threshold is highly sensitive to small changes in SHP-1 expression levels during cellular differentiation, were confirmed by experiment. These results combining computation and experiment reveal that ligand discrimination by T cells is controlled by the dynamics of competing feedback loops that regulate a high-gain digital amplifier, which is itself modulated during differentiation by alterations in the intracellular concentrations of key enzymes. The organization of the signaling network that we model here may be a prototypic solution to the problem of achieving ligand selectivity, low noise, and high sensitivity in biological responses.

## Abstract 2

Computational modeling provide a model which accounts for the high sensitivity, ligand selectivity, and low noise in TCR signaling. Several predictions of the model are supported by experimental data.

## Introduction

The functions of the adaptive immune system are regulated by intracellular signals arising from the interaction of clonally distributed, somatically generated receptors on T or B lymphocytes with antigens derived from invading infectious organisms [1,2]. The antigen receptors (T cell receptors or TCRs) on most conventional CD4^+^ and CD8^+^ T lymphocytes recognize short peptides extracted from pathogen proteins and displayed on cell surfaces in association with integral membrane proteins encoded by the major histocompatibility complex (peptide–MHC molecule ligands or pMHCs) [3]. Because the cellular machinery that creates pMHCs does not distinguish in most cases between pathogen proteins and host proteins, the surface of a cell that is being scanned by TCRs is typically a mosaic of self- and foreign-pMHC ligands [4]. This imposes a critical task on the T-cell recognition and intracellular signaling machinery, which is to avoid triggering functional responses to the highly abundant self-pMHCs while fostering rapid, highly sensitive, and specific responses to low densities of non-self-pMHCs on the same membrane. One major factor contributing to this discrimination by mature T cells is the elimination during thymic development of many immature cells possessing TCRs that are highly reactive with self-pMHCs [5,6]. However, this cellular selection itself depends on the capacity of the TCR to make fine distinctions between closely related pMHC structures when transducing signals that regulate cell survival and differentiation—distinctions that also must be made by mature, post-thymic T cells.

Two models have been put forward to account for the exquisite discrimination capacity of T cells. The first model is based on the idea that agonist pMHCs capable of functional T-cell activation induce a specific conformational change in the TCR complex [7–9]. The second model suggests that the signaling machinery of the T cell employs kinetic thresholding based on the lifetime of pMHC–TCR complexes to discriminate agonist pMHCs from non-agonist pMHCs [10–12]. Two experimental observations cannot be explained by the former model. First, among all the X-ray crystallographic structures of TCRs in complex with different pMHC ligands [13,14], none have displayed a change in conformation that is specific for an agonist pMHC in comparison to a non-agonist pMHC. Some investigators have proposed that a conformational change takes place in the signaling CD3 or ζ chains associated with the αβ ligand-binding subunits of the TCR [7–9,15,16], but the structures of these subunits in combination with the TCR remain to be solved, and convincing evidence for this hypothesis has yet to be reported. Second, and more significantly, the potency of pMHCs in activation of T cells endowed with a particular TCR is modulated during intrathymic differentiation [17]. Developing T cells (thymocytes) signal and respond functionally to self-pMHCs that are non-agonists for T cells in the periphery [18,19]. Hence, T cells with a given TCR can respond differently to the same set of pMHCs. This observation challenges explanations of ligand discrimination in T-cell activation on the basis of pMHC-specific conformational changes in TCRs, because responses to a given ligand differ in immature and mature cells despite the identity of the antigen-receptor structure.

In the second model, the signaling machinery of the T cell employs kinetic thresholding based on the lifetime of pMHC–TCR complexes to discriminate agonist pMHCs from non-agonist pMHCs [20,21]. The kinetic-proofreading concept, as first detailed for T cells by McKeithan [10] and then elaborated in several later variants of this original model [11,22,23], postulates that small differences in the longevity of pMHC–TCR associations are amplified into large differences in downstream signaling output by a signal-transduction pathway with many steps, each of which requires continued ligand–receptor interaction to occur. Indeed, the only biophysical parameter reported in multiple studies to correlate with the quality of T-cell activation is the lifetime of the pMHC–TCR complex. Measurements based on surface plasmon resonance with soluble TCR and pMHC suggest that the dissociation rate, rather than the association rate, of the complex is most sensitive to pMHC structure and relates best with biological potency of the ligand [24]. In one study, Kersh et al. [25] reported that a single amino acid substitution converted an agonist pMHC into a weak-agonist pMHC with a 2 × 10^4^-fold reduction in biological potency, while only decreasing 5-fold the lifetime of the corresponding pMHC–TCR complex (from 10.8 to 2.3 s at room temperature). Thus, modest biophysical differences in the pMHC–TCR interaction appear to result in exquisite functional pMHC discrimination by T cells.

T cells not only show this capacity to distinguish among closely related ligand structures, but also have a response to antigen that is fast, extremely sensitive, and frequently digital in nature. A single pMHC is sufficient to trigger a calcium response [23] or cytotoxic activity [26] in primed T cells. Measurements of the early phosphorylation of the TCRζ chains [27] and of the calcium response of T cells [23,28] demonstrated that T-cell signaling occurs on a very short timescale (within as few as 15 s) after antigen-presenting cell (APC)–T-cell contact. Functional activation of T cells, after hours of contact with APC, is typically characterized at the individual cell level by an all-or-none response, whether measured as cytokine gene activation [29] or proliferation. These considerations make the classical kinetic-proofreading schemes for TCR signaling unsatisfactory because these models provide adequate ligand discrimination only at the expense of sensitivity or speed of response [12,30] and fail to account for a T cell's digital response to receptor engagement [23] (see Protocol S1 for a quantitative analysis of the limitations of classical kinetic-proofreading schemes in replicating the known features of TCR signaling).

The primary aim of the present study was to develop a detailed, quantitative model of early TCR signaling that accounts for these conjoint characteristics of a T cell's response to antigen. The model incorporates direct measurements of key concentrations of signaling molecules and of pMHC–TCR ligand interactions. Experiments conducted simultaneously with the model building revealed that T cells exhibit an unexpected property in their TCR-dependent signaling, namely a digital extracellular signal-regulated kinase (ERK) response that involves an extremely high level of input amplification. By constructing our model around a kinetic-proofreading scheme modified through the inclusion of two competing feedback pathways previously proposed to sharpen the discrimination threshold between closely related TCR ligands [27], we show how spurious activation of this explosive ERK amplifier by abundant non-agonist ligands can be prevented, while retaining sensitivity to low numbers of agonist ligands. The validity of our model was examined directly by cell-based experiments testing three predictions: the rapid increase of the signaling response time when the number of ligands is decreased, the hierarchy of antagonism in T-cell signaling, and the tunability of ligand discrimination during T-cell clonal expansion. The biological responses all fit well with the predictions of the model, providing strong support for the conclusion that this differential feedback scheme represents the core network of reactions that guide T-cell signaling responses to ligands and accounts for the characteristics of this key immune event.

## Results

### Highly Amplified, Digital ERK Responses Induced by Agonist pMHC

To develop and test a predictive model of T-cell activation, we needed quantitative measurements of the early signaling events associated with TCR engagement by pMHC. In addition, because functional data on T-cell activation show a sharp distinction between agonist ligands (that elicit T-cell responses even at low densities) and structurally related non-agonist ligands (that do not elicit such responses even at high densities), we specifically sought to identify a feature of the proximal T-cell signaling pathway that reflects this ability to discriminate, in a nearly absolute manner, between agonist and non-agonist pMHCs. We focused initially on the cascade activating the ERKs. These enzymes are members of the mitogen-activated protein kinase (MAPK) family [20,31] and are particularly attractive candidates for participating in such digital discrimination because prior studies have emphasized the importance of this pathway in both regulation of TCR signaling [27] and in functional responses [32], while other work with *Xenopus* oocytes has shown that the organization of this enzyme cascade can produce an ultrasensitive response associated with cell-fate decisions [33,34].

As a model system, we chose to examine the ERK-phosphorylation response of OT-1 CD8^+^ T cells upon activation with peptide-pulsed APCs. The transporter associated with antigen processing (TAP)–deficient lymphoma RMA-S was used as the APC because this cell line does not efficiently load self-peptides into newly synthesized major histocompatibility complex class I molecules, but does effectively present exogenously added peptide via those class I molecules that reach the cell surface [35]. Thus, peptide-pulsed RMA-S cells present a homogeneous pMHC-ligand display spanning several decades in number without an appreciable pool of co-expressed self-ligands [35]. Although there is evidence that self-recognition can synergize with contemporaneous agonist recognition in the activation of at least some CD4^+^ T cells [36], a previous study failed to demonstrate a substantial role for self-ligands in the activation of OT-1 T cells using RMA-S APC [37], and we have confirmed the latter findings (unpublished data). The absence of such self-pMHCs on RMA-S APC and the evidence against such ligands contributing to T-cell activation in this T cell–APC combination allowed us to simplify both our model of TCR signaling and the corresponding experimental studies. In addition, a monoclonal antibody (25D1.16) to an agonist pMHC ligand for the OT-1 T cells (SIINFEKL-K^b^) was available [38], permitting direct quantitation of the absolute number of agonist ligands generated at each concentration of pulsing peptide (Figure S1).

**Figure 1:**
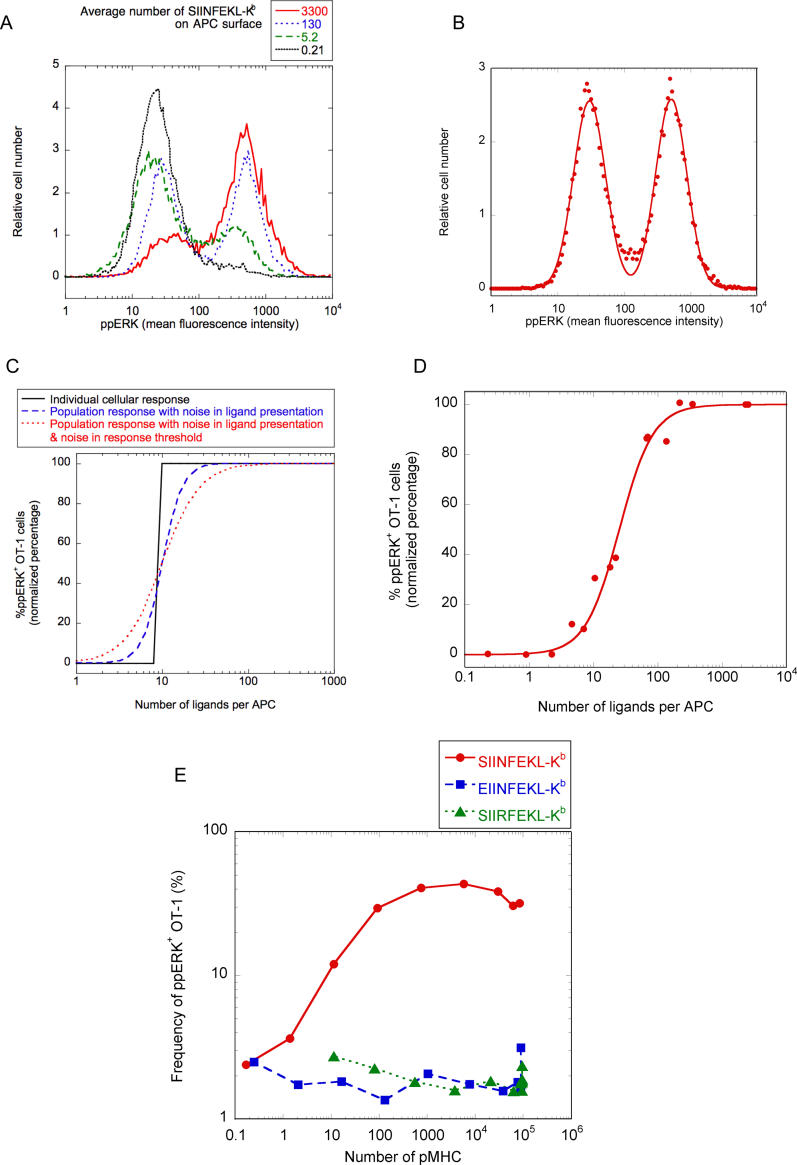
Quantitation of Speed, Sensitivity, and Specificity of the Digital ppERK Response in Naïve T Cells (A) Distribution of ERK phosphorylation (measured by flow cytometry) among individual naïve OT-1 T cells after 3 min of activation by RMA-S APCs at different levels of presentation of the agonist pMHC SIINFEKL-K^b^. (B) Fit of the distribution of ppERK responses among OT-1 T cells activated with an average of 130 SIINFEKL-K^b^ ligands on the surface of each RMA-S APC. The fit (a sum of two log-normal distributions) is statistically adequate (χ^2^ = 1.72 for 128 points, and three fitting parameters). (C) Theoretical effect of biological variation (“noise”) in ligand presentation by APCs and the responsiveness of individual T cells on the steepness of the dose response of a population of T cells. The ppERK response of an individual T cell is essentially digital (infinite Hill coefficient), but the low observed Hill coefficient (1.9) for the dose response of real T cells at a population level can be explained by taking into account the noise in ligand presentation (CV = 50%) and the possible noise in the activation threshold of the T cells (CV = 75%). (D) Experimental ppERK dose response of naïve OT-1 T cells activated for 3 min with peptide-pulsed RMA-S cells, plotted as the percentage of responding cells. The Hill coefficient measured for this dose response is 1.9 ± 0.1 (*n* = 3). The threshold for activation (midpoint) is 24 ± 4 SIINFEKL-K^b^ on each RMA-S APC. Because the T cell's surface area is three times less than that of an RMA-S cell, as few as eight SIINFEKL-K^b^ ligands may be sufficient to trigger a full ppERK response if a full surface sweep of the RMA-S membrane by the T cell is not accomplished before signaling takes place. (E) Dose response for ERK phosphorylation among naïve OT-1 T cells, after 3 min of activation by RMA-S APC pulsed with SIINFEKL peptide variants. The peptide SIINFEKL is a known agonist for OT-1 T cells, whereas EIINFEKL and SIIRFEKL are non-agonists. The percentage of responding cells is plotted as a function of the number of peptide-K^b^ ligands presented on the surface of each RMA-S APC.

Using RMA-S APCs with calibrated numbers of ligands to activate T cells, the ERK-phosphorylation response was measured by intracellular staining combined with flow cytometry. We found that the dual phosphorylation of ERK necessary for the activity of this kinase can be detected in an individual T cell when as few as ten agonist SIINFEKL-K^b^ ligands are presented on average by the APCs, with 10% of T cells showing a robust response after 3 min of T cell–APC contact (Figure 1A). These studies also revealed a previously unappreciated aspect of the T-cell ERK-signaling response: after 3 min of contact with APCs, the pattern of staining is strictly bimodal, i.e., the ERK response of T cells is essentially digital. Control experiments confirmed that the intracellular staining protocol we used was capable of detecting doubly phosphorylated, kinase-active ERK (ppERK) levels within T cells that were lower or higher than the fixed level seen among the responding cells in Figure 1A (Figure S2), indicating that the quantitatively constant nature of this signaling response is an inherent property of the T cells and not an artifact of the measurement technique. This bimodal distribution could be fitted as a sum of two discrete log-normal distributions, indicating that the individual cell ppERK response is switch-like with a nearly infinite Hill coefficient (Figure 1B). We also measured the number of ERK molecules phosphorylated during T-cell activation, using purified phosphorylated ERK for calibration. This analysis showed that the digital response of a T cell is macroscopic with the phosphorylation of 100,000 ERK proteins (Figures S2 and S3), revealing that the ERK pathway in T cells acts as a high-gain digital amplifier.

In contrast to the single-cell results, the dose-response curve for ERK activation of a population of OT-1 T cells can be fitted with a Hill coefficient of 1.9 ± 0.1 (*n* = 3) and an EC_50_ = 24 ± 4 (*n* = 3) SIINFEKL-K^b^ per RMA-S (Figure 1C). The apparent discrepancy between the infinite Hill coefficient determined at the individual cell level and the shallower dose response measured at the population level can be understood by taking into account the distribution of ligand densities on APCs and variations in responsiveness among individual T cells (“biological noise”) (Figures 1D and S1; Protocol S2). Based on these data, the average threshold for the digital ppERK response in OT-1 T cells is 24 SIINFEKL-K^b^ presented per RMA-S. Because the surface area of naïve OT-1 T cells is three times less than the surface of the RMA-S used as APCs in our experiments, the absolute threshold to trigger the phosphorylation of 100,000 ERK molecules within 3 min of T cell–APC contact may be as few as eight SIINFEKL-K^b^ ligands.

This ERK-phosphorylation response of OT-1 T cells is also specific. When non-agonist peptide variants (such as EIINFEKL or SIIRFEKL), as defined by functional response measurements, are presented on the surface of the APC, no phosphorylation of ERK above the background could be detected, even with 1 × 10^5^ pMHCs per APC (Figure 1E). Moreover, ERK phosphorylation in T cells after 3 min of activation also correlated with the functional specificity of activation when assayed by CD69 upregulation, interferon gamma (IFNγ) expression, or cytotoxicity (Figure S4). Because the only known differences between SIINFEKL-K^b^ (agonist) and the non-stimulatory EIINFEKL-K^b^ or SIIRFEKL-K^b^ ligands for the OT-1 TCR are the lifetimes of the pMHC–TCR interactions (31.5, 10.7, or 6.3 s, respectively, at room temperature [39]), our data indicate that modest differences in ligand–receptor interaction are translated into robust discrimination by the digital ppERK response of a T cell's signaling machinery.

### Model of the Early Events in T-Cell Activation

The large and rapid signal amplification associated with ERK phosphorylation in T cells, coupled with a capacity of these cells to discriminate over four orders of magnitude of ligand density between two pMHCs that differ in TCR-binding lifetime by less than 5-fold, raises major questions about how this can be accommodated by traditional kinetic-proofreading schemes. Superficially, it would seem that the system should be extremely sensitive to noise, with even very poor ligands for the TCR eventually “sneaking through” [40] and causing the digital ERK response to be activated. To deal with this problem, some form of filtration or noise suppression is needed, which in signal processing would typically be mediated by a negative-feedback system [41]. Recently, the SH2 domain-containing tyrosine phosphatase (SHP-1) has been shown to play such a role in TCR signaling [27,42], and some models of TCR discrimination have incorporated this feedback to limit responses to high levels of weakly binding ligands [23,43]. However, such negative feedback alone would also act to diminish the sensitivity of the system to otherwise agonist (stimulatory) ligands. The discovery of a positive-feedback loop involving ERK-1 that protects TCRs from the inhibitory effects of SHP-1 [27] provides a possible solution to this dilemma involving sensitivity. However, because no explicit model has yet tested whether the combination of these two divergent feedback pathways with a proofreading-based scheme would quantitatively account for the key characteristics of TCR signaling, we set out to construct such a model and test its predictive capacity.

**Figure 2:**
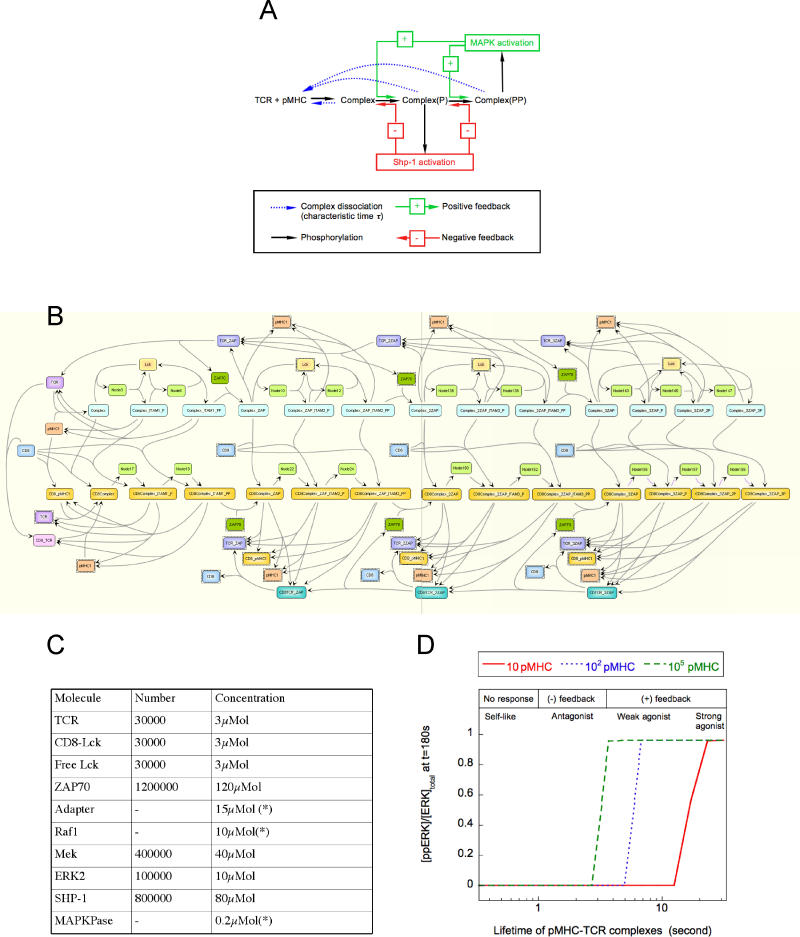
Computer Model of the Early Events of T-Cell Activation (A) Sketch of model. Differential positive-/negative-feedback loops are added to a kinetic-proofreading scheme of pMHC–TCR interaction. At early times, phosphorylated TCR complexes activate SHP-1 (a tyrosine phosphatase), which provides a negative-feedback effect by dephosphorylating components within the TCR complex. Upon TCR engagement by an agonist-quality ligand, but with a time delay, the MAPK (ERK) cascade is activated and provides a positive-feedback effect by protecting the TCR complex from binding and dephosphorylation by SHP-1. (B) Explicit model of core module of the early events of TCR signaling (see Figure S6 for an expanded view of the model). (C) Table of the number and corresponding cytoplasmic concentrations of the signaling components involved in the model. An asterisk indicates molecules whose number and concentration are estimated. (D) Output of the computer simulation. After 3 min of simulated time, the TCR signaling machinery produces a sensitive and specific ppERK response. There is also a sharp transition in the ppERK response depending on the quality of the pMHC ligands (as measured by the lifetime, *τ*, of their interaction with TCR). Four categories of ligands can be defined from the simulation. For pMHCs whose *t* is above 15 s, a complete ppERK response is obtained with as few as ten ligands; these are the strong agonists. For pMHCs whose *τ* is between 3 and 15 s, a ppERK response is obtained when sufficient numbers of ligands are present; these are the weak agonists. pMHCs whose *τ* is below 3 s fail to trigger a ppERK response; these are non-agonists. Finally, because different combinations of feedback control are triggered by each category of ligands, ligands whose *τ* is below 1 s do not trigger negative feedback efficiently. These may constitute the majority of self-ligands, preventing self-recognition from depressing responses to full agonists [69].

In Figure 2A, we present a simplified block diagram summarizing the key kinetic components of our model. Interaction of a pMHC with a TCR yields successive steps of phosphorylation of the TCR complex (e.g., of the associated CD3/ζ chains via activation of the Src family kinase Lck). Dissociation of the pMHC–TCR complex is assumed to permit the rapid dephosphorylation of TCR-complex components by a highly abundant active phosphatase (e.g.*,* CD45 [44]), which is not explicitly simulated here. Kinetic proofreading of pMHC–TCR interactions is based on the assumption that phosphorylation requires pMHC–TCR contact and that dephosphorylation rapidly reverses these events upon ligand dissociation owing to the action of abundant phosphatases. The phosphorylated TCR complexes can activate two divergent pathways. Beginning shortly after TCR engagement occurs, the phosphatase SHP-1 is tyrosine phosphorylated by active Lck [45]. The resulting pSHP-1 binds stably to Lck-containing TCR complexes via interaction with the kinase's SH2 domain. This docked SHP-1 becomes enzymatically activated upon further tyrosine phosphorylation in the TCR complex, leading to dephosphorylation of the Lck, CD3/ζ, and associated ZAP-70 kinase components of the signaling complex [27,42]. This mechanism of action permits pSHP-1 to act as a spreading negative feedback by decorating unengaged Lck-containing TCR complexes and quickly deactivating the receptor when ligand engagement initiates phosphorylation events within that complex [46].

At slightly later times after TCR engagement, TCR complexes can proceed to full phosphorylation and trigger a kinase cascade activating ERK. Active ERK, in turn, acts as a positive feedback by serine phosphorylation of the Lck in TCR complexes, a biochemical modification that prevents the kinase from binding to pSHP-1 [27]. Hence, our model network for TCR signaling can be summarized as a kinetic proofreading of the pMHC–TCR interactions, triggering the MAPK cascade as a high-gain digital amplifier, with a rapid-onset analog SHP-1-mediated negative feedback and a slower digital ERK-1-dependent positive feedback modulating the triggering threshold. The evidence for signal spreading that we have reported previously [46], in concert with the digital nature of the ERK response, enables this counter-intuitive arrangement of an early arising negative feedback and delayed positive feedback to support effective signaling.

For the purpose of comparing computer simulations with experiments, we implemented explicit chemical reactions using parameters derived as much as possible from direct measurements rather than fitting, although the latter was necessary for many of the enzymatic rates that have not been measured in a cellular context (Figures 2B, S3, and S5; Protocol S3). The only quantitative parameter distinguishing different pMHC ligands in our model is the lifetime of their interaction with TCR. The expression levels of signaling molecules were determined by quantitative immunoblotting or flow cytometry and their concentration calculated based on the measured cytoplasmic volume of naïve T cells (15 fl) (Figures 2C and S5). These measurements underscore the fact that T cells contain large concentrations of signaling molecules (>3 μMol), which is consistent with the high speed of response and also limits the impact of stochastic behavior on the chemical reactions. We therefore assumed that diffusion kinetics were not limiting and used a stirred-cell model in our simulations.

The macroscopic clustering and spatial reorganization of proteins in the immunological synapse is not required for the early rapid signals we are assessing here [20,47], and hence, the correlation of the formation of this organized multiprotein structure with effective T-cell activation [47] is not inconsistent with our assumption. JDesigner software [48] was used to define the biochemical network for TCR signaling (see Figure S6 and Protocol S4 for a complete description of the model and Protocol S3 for a complete description of the kinetic parameters used in our experiments and their origin). The computer modeling of this network involved solving a set of deterministic differential equations with a Rosenbrock formula of order 2, implemented using Matlab (see Protocol S4).

Solving our computer model for different quantities and qualities of ligands (i.e., different lifetimes of the pMHC–TCR complex) shows how the competition between positive and negative feedbacks defines a digital threshold of T-cell activation in terms of the dynamics of this ligand–receptor interaction. Figure 2D presents the simulated ppERK response after 3 min of exposure to different numbers of ligands of different receptor-binding lifetimes. This response is nearly digital with a sharp threshold at a pMHC–TCR lifetime of 3 s, comparable to the experimentally reported threshold in pMHC–TCR complex lifetime for agonist activity, extrapolated to 37 °C (see Protocol S3). Hence our model shows almost absolute discrimination with respect to the quality of pMHC–TCR ligand interaction, while also showing both fast kinetics and sensitivity to a few agonist ligands.

### Testing Three Predictions of the Differential Feedback Model of T-Cell Signaling Control

#### Lengthening of the ppERK response time at low ligand densities

In our model, the MAPK cascade of concatenated kinase phosphorylations amplifies sparse input signals (the output of the kinetic proofreading of pMHC–TCR interaction) to yield a robust ppERK response (Figures S7 and S8): indeed ten ligands were shown to drive the phosphorylation of 100,000 ERK molecules in T cells (see Figure 1A). One hallmark of such a kinase cascade is the digital response of ERK phosphorylation (i.e., large Hill coefficient at the cellular level [see Figure S8C]), which we have confirmed experimentally (see Figure 1B). A key predicted feature of such a response scheme is the nonlinear lengthening of the response time at low ligand densities (see Figure S8B and S8D).

**Figure 3:**
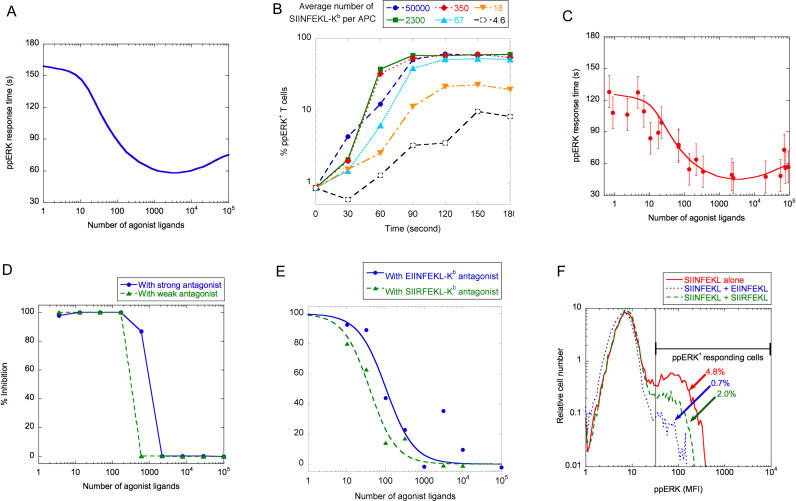
Experimental Test of Two Predictions of the Computer Simulation of the Early Events in TCR Signaling (A–C) Characteristic response time for ERK phosphorylation. The characteristic time of the ERK-phosphorylation response was derived by computer simulations of TCR signaling for increasing numbers of agonist ligands (whose lifetime of interaction with the TCR is set at 18 s) (A). This timescale diverges in a nonlinear fashion when the number of agonist ligands is decreased. We then systematically measured the kinetics of the ppERK response of naïve OT-1 T cells upon activation with RMA-S APCs presenting different numbers of SIINFEKL-K^b^ (B) and derived the characteristic time of response using a generic sigmoidal fit. The divergence of this timescale as the number of agonist ligands is decreased (C) is characteristic of kinase cascades acting as digital filters (A). (D–F) Comparison of antagonism in T-cell activation in computer simulations and experiments. (D) Computer simulation of antagonism. We simulated the ppERK response of T cells upon activation with increasing numbers of agonist ligands (whose interaction with the TCR has a lifetime of 18 s) in the presence of 30,000 non-agonist ligands (the two putative antagonist ligands being tested have TCR-interaction lifetimes of 1.7 s [weak antagonist] and 3 s [strong antagonist], respectively). The presence of a large number of sub-threshold ligands inhibits the agonist-induced ppERK response of T cells. The inhibition is calculated as the ratio of the ppERK response in T cells activated with agonist and antagonist together as compared to the ppERK response seen using the agonist alone. This hierarchy of antagonism in early T-cell responses is consistent with the graded activation of SHP-1-mediated negative feedback associated with signaling by sub-threshold ligands. (E) Experimental test of antagonism. Naïve OT-1 T cells were activated with RMA-S APCs pulsed with an increasing amount of agonist SIINFEKL peptide and an excess of EIINFEKL or SIIRFEKL peptides. (F) Experimental ppERK response of OT-1 T cells upon activation with RMA-S APCs presenting 25 agonist SIINFEKL-K^b^ ligands with or without 30,000 antagonists (SIIRFEKL-K^b^ [weak antagonist] or EIINFEKL-K^b^ [strong antagonist]).

To examine whether this behavior seen in the simulations (Figure 3A) was characteristic of the biological system, we systematically measured the kinetics of ERK phosphorylation in T cells exposed to different numbers of agonist ligands (Figure 3B). In qualitative agreement with the model (see Figure 3A; Protocol S5), the time delay before the digital ERK response increased dramatically as the number of ligands was decreased towards threshold levels (Figure 3C). There was also a second, less-pronounced, slowing-down of the kinetics of ERK phosphorylation at high levels of presentation, an effect that the model indicates arises from a rapid and massive activation of the negative feedback that limits the efficiency of triggering of the MAPK cascade at high (>1 × 10^4^) agonist display. In the case of non-agonist ligands, a similar temporal imbalance in favor of negative feedback, even at modest ligand levels, abrogates the activation of the MAPK cascade and allows the suppressive regime to dominate at all pMHC densities, preventing effective responses. Although the simulation results and experimental data fit well qualitatively, there was a systematic discrepancy of 13 s in response time that could not be resolved by parameter adjustment without losing other predictions of our simulation. More detailed modeling (in particular by taking into account membrane protein pre-clustering) or more accurate measurements of the relevant kinetic and expression parameters at physiologic temperature may help eliminate this modest kinetic discrepancy.

#### Hierarchy of antagonism in T-cell signaling

Our computer simulation also enabled us to probe the role of SHP-1 negative feedback in setting the threshold of ligand discrimination. Given the “explosive” responsiveness of the MAPK cascade, T cells must activate a negative feedback that is tuned to the strength and quantity of ligands, to blunt spurious activation with large quantities of low-affinity ligands while allowing sensitive responses towards small quantities of more strongly binding complexes. Negative feedback mediated by SHP-1 has previously been shown to be responsible for TCR antagonism [27], a phenomenon in which simultaneous exposure of a T cell to a large quantity of sub-threshold ligands and a small, otherwise stimulatory, number of agonist ligands results in blunting of the expected response [49,50]. The present quantitative model predicts that TCR antagonism has a counter-intuitive characteristic: the more closely the lifetime of a TCR–non-agonist complex approaches the threshold needed for full signaling, the more strongly this ligand will antagonize T-cell activation by agonists. That is, better binders will actually be better inhibitors until a bifurcation point is reached and they become overtly stimulatory ligands themselves.

This hierarchy of antagonism can be seen in the results of Dittel et al. [46], who reported greater TCRζ phosphorylation by more potent antagonists. This effect is shown in Figure 3D, in which we present a computer simulation of the ppERK response of our TCR signaling model for increasing numbers of agonist pMHCs (whose TCR-binding lifetime is 18 s) in conjunction with 3 × 10^4^ antagonist pMHCs (whose TCR-binding lifetimes are either 3 s or 1.7 s). We examined this prediction experimentally by measuring the OT-1 ERK response to RMA-S cells bearing an increasing amount of agonist SIINFEKL-K^b^ with or without a large number (3 × 10^4^) of non-agonist ligands on the same cell membrane (Figure 3E and 3F). EIINFEKL-K^b^ antagonized more effectively than SIIRFEKL-K^b^, consistent with the fact that the former ligand forms a longer-lived complex with OT-1 TCR than does the latter (10.7 s versus 6.3 s at room temperature [39]), in accord with the expectations of the model. The slight decrease in the ppERK level among cells responding in the presence of antagonist is also seen with cells stimulated with very low densities of agonist alone and appears to arise from the 3-min assay point capturing these cells prior to their achieving maximum ppERK levels. This slower rise to the maximum at low effective ligand densities is predicted by our model (see Figure 3A).

#### Flexibility in ligand discrimination for T cells undergoing differentiation

A third general prediction of our computer model is that the precise positioning of the kinetic threshold between agonist and non-agonist ligands for a particular TCR is set by the dynamics of the competition between positive- and negative-feedback loops, which in turn is highly sensitive to modest changes in the intracellular concentration of key components such as SHP-1. Thus, we predicted that T cells could alter their discrimination threshold by small changes in the concentration of these molecules during differentiation.

Consistent with this expectation, analysis of the TCR signaling response of activated OT-1 T cells revealed that these cells transiently demonstrated ERK responses to EIINFEKL-K^b^ 5 or 6 d after the initial activation, whereas naïve OT-1 (OT-1)_naïve_ and activated OT-1 T cells rested for 11 d (OT-1)_day 11_ were strictly unresponsive towards this ligand (Figures S9 and S10). An assessment of protein concentrations in these cells revealed a substantial (>10-fold) decrease in the concentration of SHP-1 relative to other measured key signaling molecules in (OT-1)_day-5_ T cells as compared to (OT-1)_naive_ or (OT-1)_day-11_ T cells (see Figure S9). The model suggested that the acquisition of responsiveness to a ligand showing poor TCR-binding characteristics, as we saw for (OT-1)_day-5_ T cells, could be accounted for by this relative diminution in SHP-1 levels. Simulations also predicted that the selective decrease in SHP-1 should yield a peculiar dose response to the low-affinity ligand, with a measurable ppERK response at moderate ligand concentrations, followed by a rapid loss of this signaling response as ligand density increases. This is because the decreased SHP-1 levels slow down the functioning of the negative-feedback pathway in response to moderate levels of weak ligand, allowing activation of ERK; at high levels of presentation, the pace of the SHP-1-mediated negative feedback is accelerated and overrides the delayed activation of ERK. The same change in SHP-1 level was predicted to have no detectable effect on the dose response to a strong agonist.

**Figure 4:**
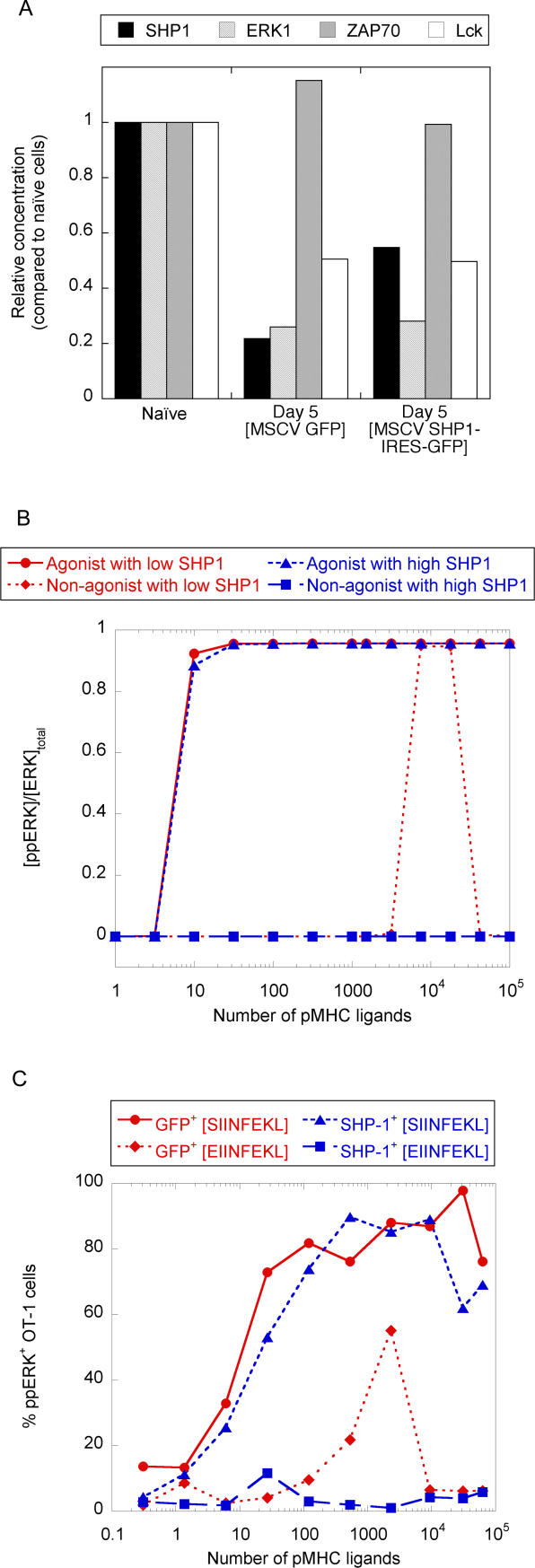
Experimental Verification of the Predicted Role of Small SHP-1 Concentration Changes in Altering Ligand Discrimination by OT-1 T Cells (A) Concentrations of signaling molecules in OT-1 T cells 5 d after activation and infection with MSCV retrovirus in vitro (day 5). These concentrations are normalized using the corresponding concentrations in the unstimulated naïve state (day 0). (B) Computer simulation of the responsiveness of T cells at day 5 after activation with the SHP-1 level set to that seen in the naïve state. The agonist pMHC is set to bind TCR with a characteristic time of 18 s and the non-agonist pMHC is set to bind TCR with a characteristic time of 3 s. (C) Elimination of the response of day-5 activated cells to EIINFEKL/K^b^ by expression of additional SHP-1. OT-1 T cells were infected with MSCV retrovirus expressing EGFP (control) or SHP-1/IRES/EGFP, and the ppERK response of infected OT-1 T cells to peptide-pulsed RMA-S was tested on day 5 after initial activation.

To test these predictions experimentally, agonist-activated OT-1 T cells were infected with a retrovirus encoding EGFP only or encoding both SHP-1 and EGFP as a bicistronic mRNA. Five days after activation, the control infected OT-1 T cells showed the expected selective decrease in SHP-1 concentration, though the decrease was of smaller magnitude than typically seen with uninfected cells and the corresponding gain in reactivity to weak ligands was less pronounced. Infection with the SHP-1-encoding virus restored the SHP-1 concentration to a level similar to that found in naïve cells (Figure 4A). As the model predicted (Figure 4B), (OT-1)_day-5_ T cells infected with EGFP-expressing retrovirus responded to EIINFEKL-K^b^, but only at intermediate ligand densities, while cells infected with SHP-1/IRES/EGFP-expressing mouse stem-cell virus (MSCV) selectively lost the EIINFEKL-K^b^ response without any associated loss of sensitivity to activation by the full agonist SIINFEKL-K^b^ (Figure 4C). These data demonstrate that ligand discrimination is not “hard-wired” into the affinity or structural match between a particular TCR and its ligand, but is modulated by differentiation-related changes in the stoichiometry of components of the signaling network downstream of the receptor.

## Discussion

An extensive literature on the intracellular signals triggered by pMHC-ligand engagement of the TCR suggests that the response is rapid, sensitive, and highly discriminatory. In this study, we have documented another key feature, namely the digital, highly amplified ERK response that occurs at short timescales (<3 min) but correlates with functional responses at >1 h post-TCR engagement. This finding raised a fundamental issue: how can T cells trigger such an “explosive” response while maintaining the specificity of ligand discrimination? In an attempt to construct a model that accounted simultaneously for all four key characteristics of TCR signaling in response to ligand engagement, we combined two recently reported opposing feedback modules [20,27] with a core scheme based on kinetic proofreading [10,11,30,43]. Using realistic kinetic parameter sets for computer simulation of the signaling cascade downstream of TCR engagement, our model yielded an output that had the striking characteristic of a sharp transition in ligand agonist functionality at TCR-binding lifetimes corresponding to those measured in several different T-cell systems [21]. We showed that this transition is also consistent with the very large (1 × >10^4^) shift in potency of pMHC ligands that differ by only a few fold in their binding lifetimes. Our modeling suggests that the sharp threshold for pMHC-receptor lifetimes yielding agonist responses originates from the distinct kinetic characteristics of the phosphatase-mediated negative feedback that suppresses signaling by weak ligands and the ERK-mediated positive feedback that is induced effectively only by more avid ligands of the TCR.

We built upon the observations of Stefanova et al. [27] in constructing a model in which SHP-1 mediated inhibition begins to function quickly upon TCR engagement, but scales in an analog way with input. In contrast, the ERK response was modeled as delayed but (as newly documented here) digital in nature. This combination allows TCR activity induced by a large number of weak ligands to be constantly repressed by a proportional negative feedback that has enough time to quench upstream signals before they reach the limit necessary to trigger the ERK response. Both modeling and experiment confirm that the ERK response is increasingly delayed in onset as the duration of pMHC–TCR binding decreases. In contrast, more strongly binding ligands, though also inducing an initial SHP-1 inhibitory response, override the limited nature of this negative feedback early after ligand engagement by quickly triggering the highly amplified ERK digital response. The magnitude of this ERK activation then prevents inhibition of those TCR not yet inactivated by the gradually rising pSHP-1 levels, permitting effective downstream signaling through diverse pathways that impinge on genes involved in T-cell differentiation. The latter expectation of a transient recruitment of pSHP-1 to agonist-engaged TCRs and the generation of an abortive proximal tyrosine-phosphorylation response in T cells exposed to an agonist when the ERK cascade is inhibited have both been observed in biochemical studies [27]. Overall, these observations provide new insight into how control circuits can be organized to suppress noise generated by large numbers of ligands while promoting highly sensitive responses to a few optimal stimuli in the same cellular context.

Our simulations enabled us to make several predictions that were verified by experiment. Most relevant to our understanding of how T cells set the threshold for discriminating between foreign and self-ligands to promote effective responses without fostering autoimmunity, we predicted that modest changes in intracellular enzyme levels would “tune” this agonist threshold during differentiation [51–53]. This prediction was confirmed in studies showing that the decreased amount of SHP-1 in T cells a few days after activation of naïve T cells accounts for a gain in response to a pMHC ligand that is incapable of stimulating naïve or resting primed cells expressing the same TCR. This sensitivity of the response-threshold position to modest alterations in the intracellular concentrations of key components of the network, particularly SHP-1, was a somewhat surprising result. Stochastic noise in the production and degradation of signaling components might be expected to produce fluctuations of a similar magnitude in key molecules [54] and hence to jeopardize accurate self-/non-self-discrimination by T cells in the periphery after the threshold is set during positive and negative selective events in the thymus.

One possible explanation for how this is avoided is that naïve T cells may have a very stable metabolism that enables them to preserve the phenotype selected for in the thymus prior to overt activation by foreign ligand. Alternatively, others have proposed that T cells can respond to tonic exposure to self-ligands by abrogating self-responsiveness while maintaining reactivity to pathogen-derived ligand [51–53,55]. Perhaps this “self-tuning” involves dynamic adjustment of the competition between positive and negative feedbacks. A third possibility is that such fluctuations do result in an occasional T cell producing potential activation signals upon self-recognition; however, in the non-inflamed state, this would lead to tolerance through deletion or anergy [56]. The danger would be if this occurred during an inflammatory response, but indeed it is just such situations that may be inciting events for autoimmunity [57].

In this same regard, the acquisition among activated T cells of overt signaling responses to variant pMHC ligands that do not evoke such responses among naïve or resting primed T cells with the same TCR is an intriguing finding whose physiological relevance is only evident in one circumstance. Hogquist et al. originally identified EIINFEKL as a peptide driving positive selection of OT-1 T cells under organ-culture conditions in which the usual display of self-peptides is limited [18]. EIINFEKL was also the strongest antagonist of OT-1 T-cell activation by SIINFEKL presented in the context of H-2K^b^ [18]. Hence, EIINFEKL-K^b^ was a ligand known to induce some positive signaling in the OT-1 thymocytes and antagonistic negative signaling in peripheral OT-1 lymphocytes. Our model and experiments enable us to hypothesize how this divergent signaling capacity of EIINFEKL-K^b^ may correlate with up/down expression of components of the TCR signaling machinery and, specifically, SHP-1 [42]. Thus, actively keeping SHP-1 levels low during early T-cell differentiation could allow self-ligands to have weak-agonist function and drive the positive selection of the T-cell repertoire, while increased SHP-1 levels would eliminate this response capacity among the mature T cells that populate the periphery [19]. The “bell-shaped” dose response induced by EIINFEKL-K^b^ using (OT-1)_day-5_ T cells has been observed in other biological systems [58,59]: our model suggests that such a nonmonotonic dose response is in fact a reflection of the activation of excess negative feedback at a high dose of weak ligands.

Why more mature T cells that have been recently activated should alter SHP-1 levels so as to regain sensitivity to stimulation by weak ligands is not yet clear, but one possibility is that activated cells use this reprogramming of the signaling threshold to take advantage of abundant self-ligands to promote further differentiation once their initial activation has been “validated” by foreign-agonist recognition. Our biological studies and simulations were both conducted in the absence of such potentially active self-pMHCs. However, a very recent study indicates that this synergy can occur in a narrow time window after previous agonist-mediated T-cell activation [36], consistent with the gain in sensitivity to fast off-rate pMHCs that has been shown here to be due to decreased SHP-1 levels in this time frame.

What are some of the potential limitations of the current model? While it has proved very successful in simulating aspects of T-cell biology that can be verified experimentally and even has correctly predicted some behaviors not previously recognized, we do not know the extent to which the simplifications we have introduced to keep the model tractable have compromised its ability to reflect T-cell physiology. First, this model lacks spatial constraints and treats the T cell as a well-stirred vessel for the first 3 min of TCR signaling. We believe this is justified, based on our quantitative analysis of naïve T cells and their contents, which reemphasized the small cytoplasmic volume of these cells and the resulting high concentrations of signaling components. For this reason, most enzymatic reactions involved in T-cell signaling are not diffusion-limited. Moreover, the spatial reorganization of membrane signaling proteins during T-cell activation that results in a mature immunological synapse [60] takes place over a substantially longer timescale than the one considered in our model [61], and initial signaling occurs prior to the large-scale protein clustering involved in the formation of this synaptic structure. This does not mean that local inhomogeneities in protein distribution in the membrane (e.g., “rafts”), or involving scaffolded protein complexes in the cytoplasm, do not influence signaling behavior.

More elaborate modeling tools that preserve spatial information ([62]; M. Meier-Schellersheim et al., unpublished data) will be needed to expand analyses of T-cell signaling. This may be particularly relevant in understanding how self-ligands can synergize with agonist pMHCs in promoting T-cell activation [36] and in better modeling the role of signal spreading among engaged and nonengaged TCR in the action of pSHP-1 and ppERK. Second, we have omitted explicit specification of a number of molecules that are well documented in the literature to affect T-cell signaling responses, such as CD45, Csk, and several adapter proteins [63,64]. However, the influence of these components on signaling was implicitly incorporated in some of the kinetic parameters (e.g., tonic dephosphorylation), and we feel this is justified by the absence of evidence that any of these components has first-order sensitivity to the quality of the pMHC–TCR ligand interaction. Third, we have introduced modifications to the kinetic parameters of pMHC–TCR interaction measured at room temperature to match the model's output to biological experiments conducted at 37 °C. Whether our approximations in this regard are accurate are not yet clear, because evidence for both linear and nonlinear effects of temperature on pMHC interactions with TCRs have been reported [9,39,65]. Finally, we have considered here only the signaling involved under conditions in which the CD8 coreceptor plays a key role in the response. This is not an absolute necessity for all TCR-mediated activation, but it is a common feature of many physiological T-cell responses including that of the OT-1 cells we used for the biological component of the present study.

Although the primary aim of this work has been to better understand how TCR signaling is regulated and contributes to the proper performance of T cells in the immune system, the results we have obtained showing how simple feedback loops operate to suppress biological noise, amplify responses, and allow flexibility of function are likely to be relevant to many other biological systems. The roles of negative and positive feedback are well documented in gene regulatory networks [66] and, in particular, in developmental systems, where they can impose irreversible state changes on the system, providing unidirectionality to differentiation events [67]. It remains to be seen whether the specific features of the opposing feedback pathways modeled here, (rapidly initiating analog negative feedback versus delayed, digital positive feedback) are critical in other biological systems. More generally, the value we document here for quantitative modeling rather than just qualitative cartoon depiction of signaling circuits, and the importance of documenting physiologically relevant signaling dynamics in simulation outputs, indicate that methods and tools for constructing models, conducting simulations, and measuring values will be increasingly critical aspects of experimental biology.

## Materials and Methods

### Computer modeling of the early events in T-cell activation

The network of the biochemical reactions taking place upon pMHC interaction with a TCR was created using JDesigner (http://www.cds.caltech.edu/˜hsauro/JDesigner.htm) and converted into a Matlab file. The resulting set of deterministic differential equations was solved with a Rosenbrock formula of order 2 implemented. Complete descriptions of the models (for the simple kinetic-proofreading schemes and for the full TCR signaling cascade) are available in Protocol S1 and Figure S6.

### Cells, peptides, proteins, and antibodies

Splenocytes and lymphocytes were isolated from H-2^b^ OT-1 TCR transgenic mice (Tacline 175, Taconic [18]) on a Rag-2^−/−^ background [68] and used directly as responding naïve OT-1 T cells. RMA-S TAP-deficient T cell lymphoma cells [35] were used as APCs. The agonist ovalbumin peptide SIINFEKL and its variants SAINFEKL, EIINFEKL, SIIRFEKL (all >95% pure) were obtained through the National Institute of Allergy and Infectious Disease Research Technologies Branch. E10 antibody against ppERK was purchased from Cell Signaling Technology (Beverly, Massachusetts, United States); MR9–4(PE) against Vβ5.2, 53–6.7(PE) against CD8α, H1-2F3(PE) against CD69, and XMG1.2(APC) against IFNγ were from BD Biosciences Pharmingen (San Diego, California, United States); K-23 against ERK1, C-19 against SHP-1, and C-14 against ERK2 were from Santa Cruz Biotechnology (Santa Cruz, California, United States); 3A5 against Lck, purified MEK, purified ppERK, and purified ZAP70 proteins were from Upstate Technologies (http://www.upstate.com). SHP-1 was purified from the lysates of *Escherichia coli* cells transformed with a SHP-1-GST-encoding plasmid (a gift from D. Nandan, University of British Columbia, Vancouver, Canada) and calibrated against pure GST. The 25D1.16 monoclonal antibody specific for SIINFEKL-K^b^ [38] was used as a hybridoma supernatant.

### Quantitation of peptide presentation

RMA-S cells were pulsed with serial dilutions of stimulating peptides for 1–2 h at 37 °C in serum-supplemented RPMI-1640 medium. Cells were washed and then stained with phycoerythrin-coupled anti-H-2K^b^ AF6–88.5 antibody, whose fluorescence was calibrated with Quantibrite beads (BD Biosciences Pharmingen) and anti-IgG beads (Bangs Laboratories, http://www.bangslabs.com). We also used the combination of anti-SIINFEKL-K^b^ 25D1.16 antibody and phycoerythrin-conjugated anti-mouse antibodies (Jackson Immunoresearch, West Grove, PA), calibrated with the anti-H-2K^b^ staining at high levels of presentation, to achieve better resolution at low concentration of pulsing peptides.

### Flow-cytometric measurement of intracellular signaling responses

T cells (5 × 10^5^) were mixed with peptide-pulsed RMA-S (2 × 10^6^), spun at 370 *g* for 5 s, and placed at 37 °C for various amount of time. T cell–APC conjugates were then separated with ice-cold PBS/2.5 mM EDTA, and fixed with 4% paraformaldehyde for 30 min on ice. Cells were then permeabilized with 90% methanol for 30 min on ice, washed twice with PBS/4% fetal bovine serum (FACS buffer), incubated with 1 μg/ml E10 in FACS buffer, and finally stained with 1 μg/ml of phycoerythrin-labeled anti-mouse immunoglobulin. Staining was immediately measured by flow cytometry (FACSCalibur, BD Biosciences Pharmingen), after gating for small cells based on forward scatter. Calculation of the percentage of ppERK^+^ cells was performed with FlowJo (Treestar, http://www.treestar.com).

### Determination of the characteristic ppERK response time

We defined the characteristic ppERK response time of a T cell as the time yielding 50% of the maximal response for a given level of presentation of SIINFEKL-K^b^.

### Flow-cytometric analysis of functional T-cell activation

T cells (5 × 10^5^) were mixed with peptide-pulsed RMA-S (2 × 10^6^), spun at 10,000 rpm for 5 s in an Eppendorf microfuge (Hamburg, Germany), and placed at 37 °C for 3 h. T cell–APC complexes were then dissociated with ice-cold PBS/2.5 mM EDTA, and stained for CD69, then analyzed by FACS. The fraction of live APC was also determined to yield a measure of the cytotoxic activity of the T cells. For IFNγ expression, T-cell activation was performed as before, with the addition of 2 μm monensin. Cells were fixed, permeabilized with FACS buffer containing 0.1% saponin, stained for IFNγ, and analyzed by flow cytometry.

### Quantitative measurements of intracellular protein levels

Intracellular protein levels were assessed by the lysis of 100,000 naïve OT-1 T cells in 1% NP-40 (Pierce Biotechnology, Rockford, Illinois, United States) with complete protease inhibitor (Boehringer Ingelheim, Ingelheim, Germany). The proteins in these lysates were separated by SDS-PAGE using an 8%–16% gel in parallel with serial dilutions of protein standards for immunoblotting calibration and then transferred to nitrocellulose membranes. After development of the blot with the relevant antibodies, a Kodak Image Station 440 was used to quantitate the bands and yield the protein content per cell. To quantitate the expression of receptors on the surface of T cells, we used standard beads (Quantibrite, BD Biosciences Pharmingen) to calibrate the antibody staining of Vβ5.2 and CD8α.

### Fit of the distribution of ppERK

The pattern of ppERK in T cells as measured by flow cytometry was fitted with the sum of two log-normal distributions, representing nonactivated and fully activated T cells. Free parameters were the modes of staining of ppERK^−^ cells and ppERK^+^ cells, and the percentage of ppERK^+^ cells. Coefficients of variation (CVs) were set at 55%, corresponding to the measured CV for the distribution of ERKs in naïve T cells.

### Overexpression of SHP-1 by retroviral infection of activated OT-1 T cells

To examine the effect of altering SHP-1 levels on signaling in response to various pMHC ligands of the TCR, we used the retroviral vector MSCV expressing either SHP-1/IRES/EGFP or just EGFP [27]. Ecotropic Phoenix packaging cells (a kind gift of G. Nolan, Stanford University, Palo Alto, California, United States) were transfected with DNA corresponding to these two viral constructs, and supernatants were collected for spin-infection of OT-1 T cells undergoing proliferation after activation with OVA-pulsed splenocytes from B6 mice, followed by culture in 7.5% T-STIM (BD Biosciences Pharmingen) [27]. The ppERK response of infected cells to peptide-pulsed RMA-S was measured after gating on EGFP^+^ cells. Estimate of intracellular levels of expression of signaling components was performed by immunoblotting using lysates corresponding to 100,000 T cells. Correction for the low percentage of infection (typically 15%) was made to estimate the overexpression of SHP-1 in MSCV(SHP-1/IRES/EGFP)–infected T cells compared to MSCV(EGFP)–infected T cells.

## Supporting Information

Figure S1 Calibration of the Presentation of SIINFEKL-K^b^ on the Surface of RMA-S APC (A) Distribution of presentation of SIINFEKL-K^b^ on the surface of RMA-S APCs, measured by staining with 25D1.16 antibody, for different concentrations of the agonist peptide SIINFEKL.
(B) Quantitation of agonist pMHC presentation on the surface of RMA-S APCs. Calibration of the fluorescence staining (mean fluorescence intensity) by the 25D1.16 antibody was performed with Quantibrite beads (BD Biosciences Pharmingen).
(C) Fit of the distribution of 25D1.16 antibody fluorescence staining on the surface of RMA-S APC pulsed with 10 nM SIINFEKL peptide. The distribution is log normal with CV = 51% (χ^2^ = 3.2 for 100 points, and three fitting parameters).
(D) CV of the distributions presented in (A) for different concentrations of SIINFEKL.
(213 KB PDF).

Figure S2 Estimate of the Absolute Number of ERK Molecules Involved in the pMHC Response of Naïve OT-1 T Cells To estimate the intrinsic ERK phosphorylation in OT-1 T cells independently of pMHC stimulation and the maximal possible response, we compared the FACS staining with E10 anti-ppERK or anti-mouse IgG1 isotype control for OT-1 cells alone, OT-1 cells activated with unpulsed RMA-S, OT-1 cells activated with SIINFEKL-pulsed RMA-S cells, and phorbol myristic acetate–activated OT-1 cells (as a control for maximal response). Typically, the full ppERK response of naïve T cells to APC stimulation involves 50% of the total pool of ERK. The ppERK response that is independent of pMHC stimulation is negligible within our experimental resolution.
(221 KB PDF).

Figure S3 Quantitation of Surface and Cytoplasmic Signaling Proteins (1A and 1B) The numbers of TCR and CD8 molecules on OT-1 T cells were determined using calibrated flow-cytometric measurements.
(2A and 2B) The number of intracellular signaling proteins per cell was determined by immunoblotting using purified proteins as calibration standards. An example of the method as applied to ERK2 is presented.
(405 KB PDF).

Figure S4 Comparison of ERK Phosphorylation, IFNγ Production, and the Cytotoxic Activity of Rested OT-1 T Cells Following Activation by Peptide-Pulsed RMA-S APCs (A) ERK phosphorylation, (B) IFNγ production, and (C) cytotoxic activity. Biological responses of OT-1 T cells were measured as described in Materials and Methods for various concentrations of SIINFEKL, EIINFEKL, and SIIRFEKL peptides used to pulse RMA-S APCs. For these experiments, we used OT-1 T cells that had been previously activated 8 d prior to the assay, expanded in medium supplemented with 7.5% T-stim and 10% FCS, and rested for 2 d before restimulation.
(165 KB PDF).

Figure S5 Measurement of the Cytoplasmic Volume of Naïve OT-1 T Cells Confocal microscopy was used to determine the cytoplasmic volume of naïve OT-1 T cells. The diameter of naïve T cells is 5.6 ± 0.5 μm (*n* = 10)—hence a cellular volume of 90 fl. After measuring the volume of the nucleus, the cytoplasmic volume of T cells can be estimated to be 15 ± 3 fl. The cytoplasmic concentrations of ZAP70, MEK1, and ERK2 are 120 μm, 20 μm, and 10 μm, respectively.
(1.4 MB PDF).

Figure S6 Explicit Description of the Computer Model of the early TCR Signaling Events Our model was designed using JDesigner software. To view the model itself, download the software from http://www.cds.caltech.edu/˜hsauro/JDesigner.htm. Then download the model and open it using the JDesigner program. Boxes represent molecules or molecular complexes. Arrows represent chemical reactions (either reversible or irreversible). Boxes labeled “Node” represent active intermediates in enzymatic reactions. Dashed boxes are alias nodes (used when the same molecular species appears at many places in the model). It should be noted that two parameters must be specified before running any simulation: first, the number (i.e., concentration) of pMHC, and second, the lifetime of the pMHC–TCR complex.
(A and B) Complete model for TCR signaling, yielding fast and sensitive ligand discrimination.
(C–K) Separate modules of the biochemical network.
(13 MB PDF).

Figure S7 Three Outputs of the Computer Simulation of the Early Events in Naïve T-Cell Activation Four different pMHC ligands are tested in these simulations, with TCR-binding lifetimes of 0.3 s (representing a self-ligand involved in thymic selection of mature T cells), 3 s (antagonist), 7 s (weak agonist), and 18 s (strong agonist). These simulations were performed with 1 × 10^4^ pMHC being presented to a naïve T cell on each APC.
(A) Kinetics of phosphorylation of the adapter.
(B) Kinetics of loading of pSHP-1 onto Lck in TCR complexes. SHP-1-decorated TCR complexes cannot drive downstream signaling. Note the transient decoration of TCR by SHP-1 for strong-agonist and weak-agonist ligands: these ligands trigger a ppERK response, which protects TCR-bound Lck from further SHP-1 binding. This transient binding of pSHP-1 to components of the TCR complex has been observed in 5C.C7 T cells activated using agonist ligand [27]
(C) Kinetics of ppERK response. Only weak- and strong-agonist pMHC can trigger a ppERK response. These simulations fit well with the experimental results presented in Figure 3B.
(72 KB PDF).

Figure S8 Computer Simulation of the MAPK Module (A) Molecular scheme to simulate the MAPK cascade [33].
(B) Computer simulation of the kinetics of ppERK response in the MAPK module for different numbers of Ras–GTP ligands.
(C) Dose-response curve for the phosphorylation of ERK as a function of the number of Ras–GTP ligands. This curve could be fitted with a Hill coefficient of 13 and an EC_50_ of 5.3. In our model, the MAPK module acts as digital filter with a low threshold.
(D) Characteristic time of ERK phosphorylation in our computer simulation as a function of input Ras–GTP. Note the divergence of time to ppERK generation when Ras–GTP decreases towards the absolute threshold of response.
(220 KB PDF).

Figure S9 Modulation of Peptide-K^b^ Responsiveness of OT-1 T Cells at Three Stages of Differentiation (A) Comparison of the ppERK response of OT-1 T cells to TCR restimulation at different times after activation, proliferation, and differentiation in vitro. The response was measured after 3 min of contact with RMA-S APCs pulsed with agonist (SIINFEKL) or non-agonist (EIINFEKL) peptides. The response to the agonist is essentially the same at the three stages of differentiation. Non-agonist EIINFEKL peptide does trigger a ppERK response in day-5 T cells, but only for intermediate levels of presentation.
(B) Measurement of the concentrations of different signaling molecules in OT-1 T cells 5 d after activation in vitro (day 5), or 11 d after activation in vitro (day 11). These concentrations were measured by flow cytometry after intracellular staining and are presented after normalization by the corresponding concentrations in the unstimulated naïve state (day 0). Note that SHP-1 is significantly reduced in day-5 cells.
(301 KB PDF).

Figure S10 Functional Response of OT-1 T Cells (6 d after Initial Activation in vitro) Examples of the gain of functional responses (CD69 upregulation [A] and cytotoxicity [B]) to a narrow presentation range of EIINFEKL-K^b^ that parallel with the ppERK responses shown in Figure S9. This experiment is representative of seven experiments. In three other experiments, no acquisition of EIINFEKL-K^b^ responsiveness was observed on the particular day that was studied after activation. Neither was SHP-1 decreased relative to other signaling components in these particular experiments. This appears to reflect the narrow time window within which the discordance in signaling molecule concentrations occurs in these cultures, and which can vary by a day, or more in individual experiments, such that the phenomenon can be missed when only a single day post-activation is analyzed.
(202 KB PDF).

Protocol S1 Comparison of Kinetic Proofreading Schemes of TCR Signaling Protocol S1 presents quantitative arguments to show that classical kinetic-proofreading schemes fail to reconcile the conjoint requirements of speed, sensitivity, and specificity in T-cell activation.
(198 KB PDF).

Protocol S2 Computation of the ppERK Response of T Cells at the Population Level Based on the ppERK Response at the Individual Cell Level Protocol S2 presents a quantitative derivation of the ppERK dose response of T cells to presented ligands. The noise in the number of presented ligands and the all-or-none ppERK response of T cells are convolved to yield the final dose response of T cells. This derivation reconciles the all-or-none response of T cells as measured at the individual cell level, with the broad dose response measured at the population level.
(41 KB DOC).

Protocol S3 Biochemical Kinetic Parameters Used in the Computer Model of TCR Signaling in Naïve OT-1 T Cells Protocol S3 reports all the biochemical kinetic parameters derived from biophysical and enzymological measurements drawn from the literature. These are the parameters that we used to model the early events in TCR signaling.
(83 KB DOC).

Protocol S4 Computer Model of the Early TCR Signaling Events See Figure S6 for viewing instructions.
(257 KB XML).

Protocol S5 Reduction of the Divergence in Response Times of T Cells at Low Ligand Presentation by the Biological Variation (“Noise”) in Ligand Presentation To match computer simulation to experiment, the theoretical output for the time required to generate a ppERK response in T cells was convolved taking into account the log-normal distribution of ligand presentation on individual APCs.
(28 KB DOC).

## Abbreviations

APC: antigen-presenting cell
CV: coefficient of variation
ERK: extracellular signal-regulated kinase
IFNγ: interferon gamma
MAPK: mitogen-activated protein kinase
MSCV: mouse stem-cell virus
pMHC: peptide–major histocompatibility complex molecule
ppERK: doubly phosphorylated
SHP-1: SH2 domain-containing tyrosine phosphatase
TAP: transporter associated with antigen processing
TCR: T cell receptor
