# Supplementary material for: Modeling T Cell Antigen Discrimination Based on Feedback Control of Digital ERK Responses
Source: PLoS Biol. 2005 Oct 25;3(11):e356. doi: 10.1371/journal.pbio.0030356 (PMC1262625; doi:10.1371/journal.pbio.0030356)
Supplement: Supplementary file 1 [file pbio.0030356.sd001.pdf]

Protocol S1. Comparison of kinetic proofreading schemes of TCR signaling

Kinetic proofreading [KP], the standard scheme proposed to explain T cell specificity, is not sufficient to understand the speed and sensitivity of ligand-induced signaling

All the non-allosteric models proposed to explain T cell ligand discrimination are based on translating modest kinetic variations in pMHC-TCR interaction into large downstream signaling output differences. McKeithan [1] was the first to propose in detail that successive phosphorylations of the TCR-associated chains in combination with additional downstream signaling events provide a lag time between the initial binding of a pMHC to the TCR and the full activation of the lymphocyte. Dissociation of the pMHC from the TCR was presumed to prevent completion of any signaling steps in the cascade that had not yet occurred, providing a temporal filter that limits the ability of ligands that bind poorly to the TCR to induce functional responses.

Because of the dominant influence of KP models in TCR signaling simulations, we re-examined KP schemes to test whether they could achieve the required speed and sensitivity as well as specificity. First, a simple two-step proofreading scheme was implemented in which the TCR complex undergoes two irreversible modifications (e.g., phosphorylation) upon binding to pMHC, with a fixed characteristic timescale $\omega_{proofreading}$ (Figure P1.A [figure at end of protocol]). We simulated the three-minute response of this simple biochemical model for two typical combinations of ligand-TCR (the agonist pMHC binds to TCR with a lifetime of 18s, while the non-agonist pMHC binds with a lifetime of 3s) for different numbers of ligands. Defining the output of this biochemical model as the number of doubly-phosphorylated TCR complexes (which is the minimum condition necessary for the complex to interact with the next kinase in the signaling cascade), one can show that below 7 pMHCs, the output is null, between 7 and 300 pMHCs, only the agonist yields a response, and above 300 pMHCs, both agonist and non-agonist ligands produce a response (Figure P1.B). Hence, a two-step kinetic proofreading scheme achieves discrimination between these agonist and non-agonist ligands (with lifetime differing by 0.8 decade) only across 1.6 decades of pMHC display.

Yet experiments show that such binding differences in ligand-TCR interaction can give rise to functional discrimination of > 3 –4 decades of ligand density [2, 3]

To improve this discrimination, one can add more proofreading steps. In Figure P1.D, we present a scheme including six irreversible steps. Its three-minute response with $\omega_{proofreading}$ of 15 seconds is null below 65 pMHC, and only the agonist pMHC is stimulatory above that threshold (the non-agonist ligand never completes the proofreading steps). Although discrimination based on the number of pMHC yielding a response specific now spans 3.2 decades, approaching the level seen in functional tests, such a six-step proofreading scheme requires nearly 2 logs more agonist pMHCs to trigger a minimal response than experiments show are necessary [4, 5].

To optimize the kinetic proofreading and achieve response across more than four decades of agonist pMHC number while maintaining non-responsiveness to the non-agonist, we systematically tested the response of this simple biochemical model for proofreading timescales between 0.3s and 30s (Figures 1C & F). Three possible outcomes are represented and color-encoded for a three-minute simulation: (1) the model does not produce any fully-phosphorylated complex (red zone); (2) the agonist pMHC but not the non-agonist pMHC produces at least one fully-phosphorylated complex (green); and (3) both agonist and non-agonist pMHC produce a fully phosphorylated complex (yellow). A satisfactory match with T cell activation would occur if, for a given proofreading timescale, the green zone extends from 10 to 10^5 pMHC. We find that even with six proofreading steps, there is no proofreading timescale achieving the required conditions. The range of pMHC number within which agonist ligands induce a response while the non-agonist fails to do so never spans the required four decades.

Thus, there is a fundamental problem associated with classical KP schemes when modeling TCR signaling. To be able to abrogate the response of large quantities of lower affinity pMHC, such a model would have to include so many proofreading steps that the responsiveness of the biochemical network would be slow and negligible for small quantities of agonist pMHC (Figure P1.E), that is, specificity is achieved at the expense of speed and sensitivity.

An additional caveat of kinetic proofreading must be pointed out. T cell signaling response is known to be “macroscopic” even on short timescales. For example, one can

estimate the total number of calcium ions entering the cytoplasm during the first 3 min of T cell activation:

$$\int_0^{3\min} \left[Ca^{2+} \right] V_{cytoplasm} N_A \, dt = 5 \times 10^6 . s$$

where N_A is the Avogadro number and $V_{cytoplasm}$ is T cell's cytoplasmic volume ($V_{cytoplasm}$ = 15fl). This robust calcium response has been documented even for very small numbers of pMHC ligands [6]. Thus, to translate sparse signaling events associated with a few TCR-pMHC interactions to such macroscopic molecular responses, T cells must rely on substantial signal amplifications. This requirement constitutes a conceptual challenge to the molecular specificity of T cells, not addressed by standard KP schemes. The inclusion of serial engagement [7, 8] in such models, in which one pMHC sequential triggers many TCR completely, only provides an amplification of up to 100 fold, suggesting that additional intracellular amplification is necessary.

References

1. McKeithan, T.W., *Kinetic proofreading in T-cell receptor signal transduction.* Proceedings of the National Academy of Sciences of the United States of America, 1995. **92**(11): p. 5042-6.
2. Kersh, G.J., et al., *High- and low-potency ligands with similar affinities for the TCR: the importance of kinetics in TCR signaling.* Immunity, 1998. **9**(6): p. 817-26.
3. Krogsgaard, M., et al., *Evidence that structural rearrangements and/or flexibility during TCR binding can contribute to T cell activation.* Molecular Cell, 2003. **12**(6): p. 1367-78.
4. Sykulev, Y., et al., *Evidence that a single peptide-MHC complex on a target cell can elicit a cytolytic T cell response.* Immunity, 1996. **4**(6): p. 565-71.
5. Irvine, D.J., et al., *Direct observation of ligand recognition by T cells.* Nature, 2002. **419**(6909): p. 845-9.
6. Li, Q.J., et al., *CD4 enhances T cell sensitivity to antigen by coordinating Lck accumulation at the immunological synapse.*. Nature Immunology, 2004. **5**(8): p. 791-9.
7. Valitutti, S., et al., *Serial triggering of many T-cell receptors by a few peptide-MHC complexes.*. Nature, 1995. **375**(6527): p. 148-51.
8. Itoh, Y., et al., *Serial TCR engagement and down-modulation by peptide:MHC molecule ligands: relationship to the quality of individual TCR signaling events.* Journal of Immunology, 1999. **162**(4): p. 2073-80.

Figure P1: computer testing of the classical kinetic proofreading scheme of TCR-pMHC interaction

A) Two-step kinetic proofreading scheme.
B) Computer estimates of the number of doubly-phosphorylated TCR complexes formed in a 3 min simulation run of two-step kinetic proofreading, plotted as a function of the number of pMHCs presented (the proofreading timescale ω is set at 12 s in this simulation).
C) Systematic scanning of proofreading timescales for the two-step scheme. Three possible outcomes are represented and color-encoded for a three-minute run: the scheme does not produce any doubly-phosphorylated TCR complexes (black), the agonist pMHC does produce at least one doubly-phosphorylated TCR complex but the non-agonist pMHC (gray) does not, and both agonist and non-agonist pMHC produce a doubly phosphorylated TCR complex (white). The biochemical network matches the characteristics of T cell activation if the gray zone extends from 10 to 10^5 pMHC for a given proofreading timescale.
D) Six-step kinetic proofreading scheme.
E) Computer estimates of the number of doubly-phosphorylated TCR complexes formed in a 3 min simulation run of six-step kinetic proofreading, as a function of the number of pMHCs presented (the proofreading timescale ω is set at 12 s in this simulation).
F) Systematic scanning of proofreading timescales for the six-step scheme. The color encoding is the same as in (C).

In these computer models, the agonist pMHC binds TCR with a characteristic lifetime τ of 18 s and the non-agonist pMHC with a characteristic lifetime τ of 3 s.

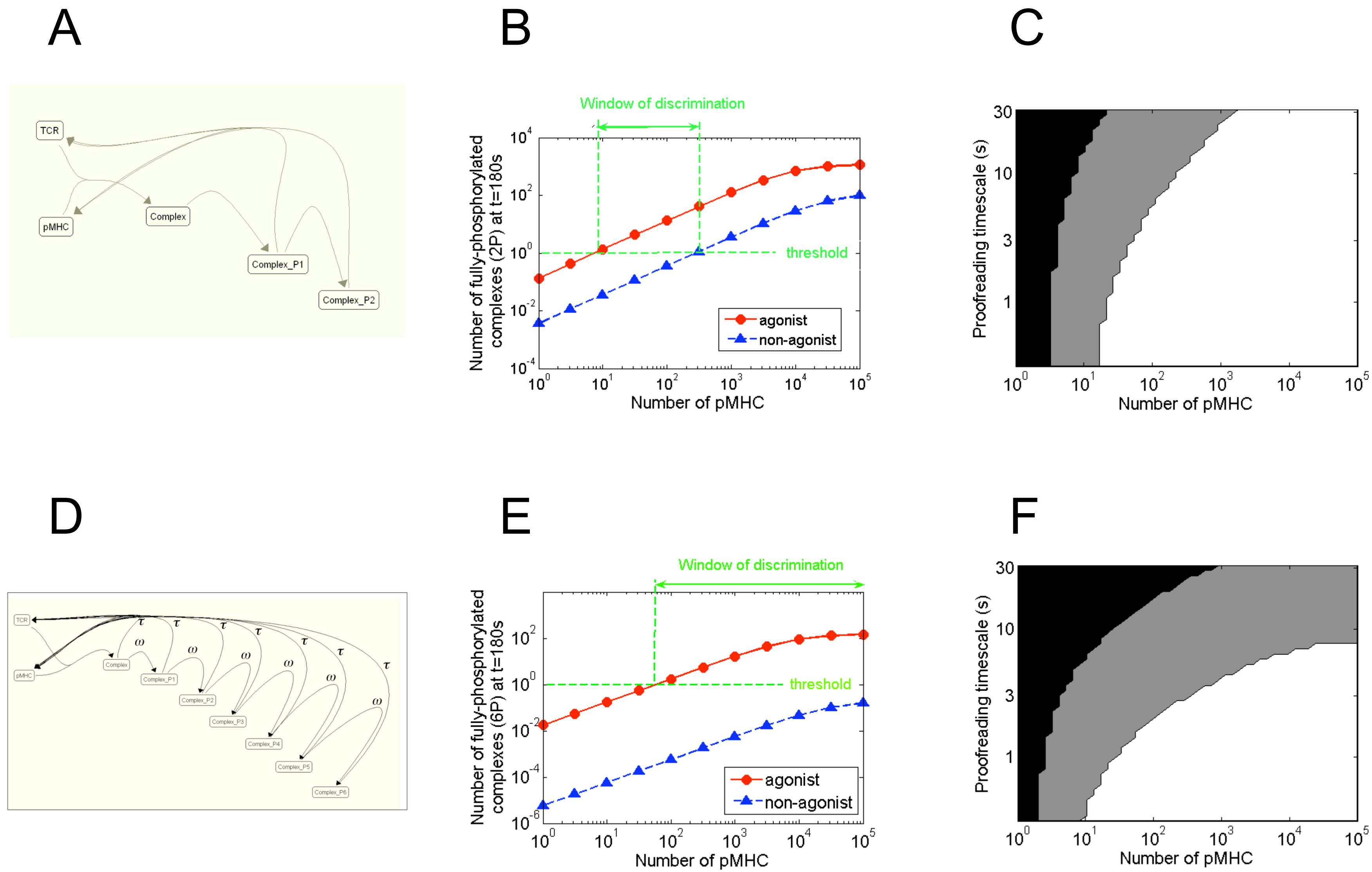
A
TCR
pMHC
Complex
Complex_P1
Complex_P2
B
Window of discrimination
threshold
agonist
non-agonist
Number of fully-phosphorylated complexes (2P) at t=180s
Number of pMHC
C
Proofreading timescale (s)
Number of pMHC
D
TCR
pMHC
Complex
Complex_P1
Complex_P2
Complex_P3
Complex_P4
Complex_P5
Complex_P6
τ
ω
E
Window of discrimination
threshold
agonist
non-agonist
Number of fully-phosphorylated complexes (6P) at t=180s
Number of pMHC
F
Proofreading timescale (s)
Number of pMHC
