# Supplementary material for: Modeling T Cell Antigen Discrimination Based on Feedback Control of Digital ERK Responses
Source: PLoS Biol. 2005 Oct 25;3(11):e356. doi: 10.1371/journal.pbio.0030356 (PMC1262625; doi:10.1371/journal.pbio.0030356)
Supplement: Supplementary file 2 [file pbio.0030356.sg001.pdf]

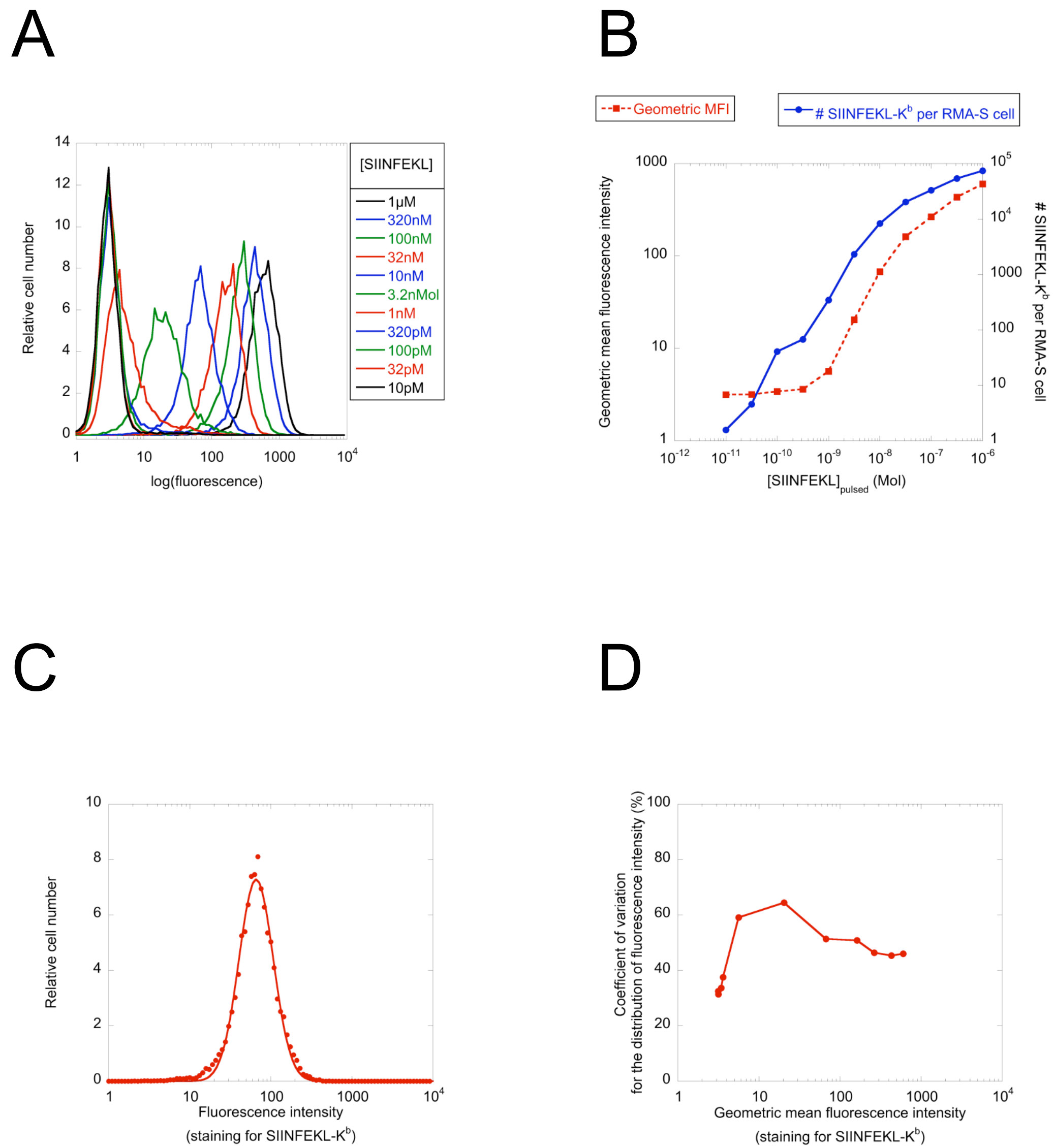
A
B
C
D
[SIINFEKL]
1μM
320nM
100nM
32nM
10nM
3.2nMol
1nM
320pM
100pM
32pM
10pM
Relative cell number
log(fluorescence)
Geometric MFI
SIINFEKL-K^b per RMA-S cell
Geometric mean fluorescence intensity
SIINFEKL-K^b per RMA-S cell
[SIINFEKL]pulsed (Mol)
Relative cell number
Fluorescence intensity
(staining for SIINFEKL-K^b)
Coefficient of variation
for the distribution of fluorescence intensity (%)
Geometric mean fluorescence intensity
(staining for SIINFEKL-K^b)
