# Supplementary material for: Modeling T Cell Antigen Discrimination Based on Feedback Control of Digital ERK Responses
Source: PLoS Biol. 2005 Oct 25;3(11):e356. doi: 10.1371/journal.pbio.0030356 (PMC1262625; doi:10.1371/journal.pbio.0030356)
Supplement: Supplementary file 3 [file pbio.0030356.sg002.pdf]

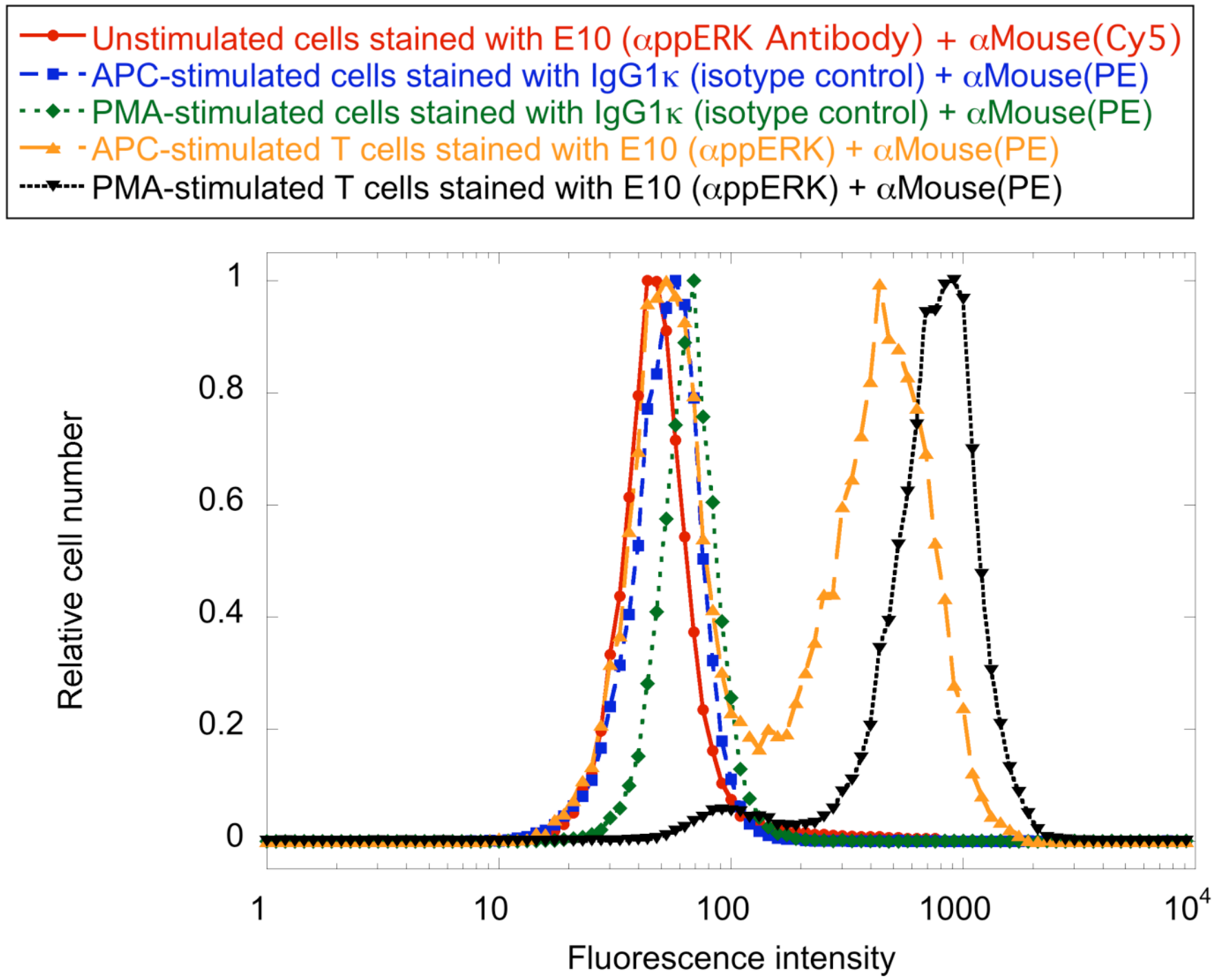
Unstimulated cells stained with E10 (αppERK Antibody) + αMouse(Cy5)
APC-stimulated cells stained with IgG1κ (isotype control) + αMouse(PE)
PMA-stimulated cells stained with IgG1κ (isotype control) + αMouse(PE)
APC-stimulated T cells stained with E10 (αppERK) + αMouse(PE)
PMA-stimulated T cells stained with E10 (αppERK) + αMouse(PE)
Relative cell number
1
0.8
0.6
0.4
0.2
0
1
10
100
1000
10^4
Fluorescence intensity
