# Supplementary material for: Modeling T Cell Antigen Discrimination Based on Feedback Control of Digital ERK Responses
Source: PLoS Biol. 2005 Oct 25;3(11):e356. doi: 10.1371/journal.pbio.0030356 (PMC1262625; doi:10.1371/journal.pbio.0030356)
Supplement: Supplementary file 4 [file pbio.0030356.sg003.pdf]

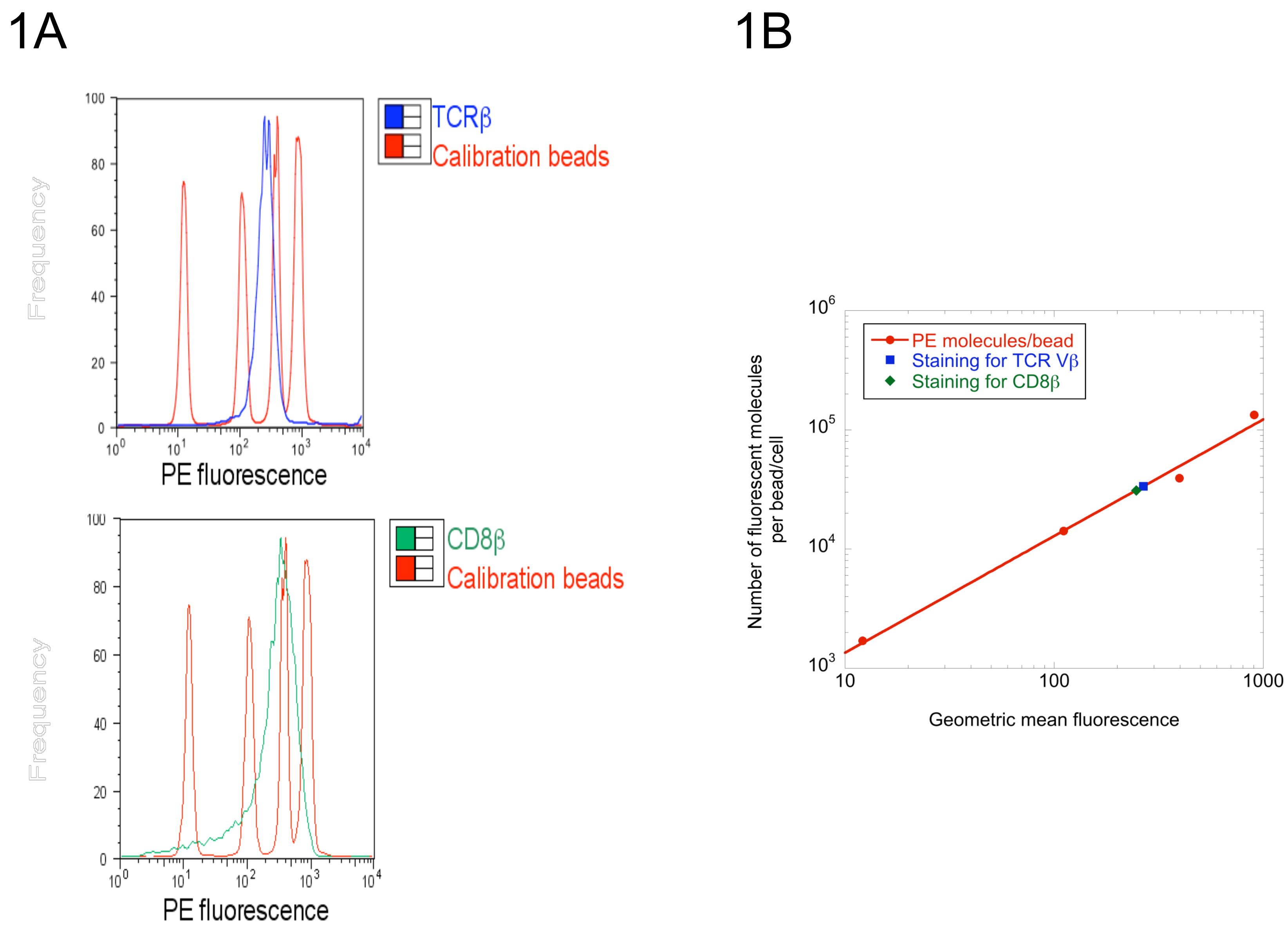
1A
TCRβ
Calibration beads
Frequency
PE fluorescence
CD8β
Calibration beads
Frequency
PE fluorescence
1B
PE molecules/bead
Staining for TCR Vβ
Staining for CD8β
Number of fluorescent molecules per bead/cell
Geometric mean fluorescence

2A

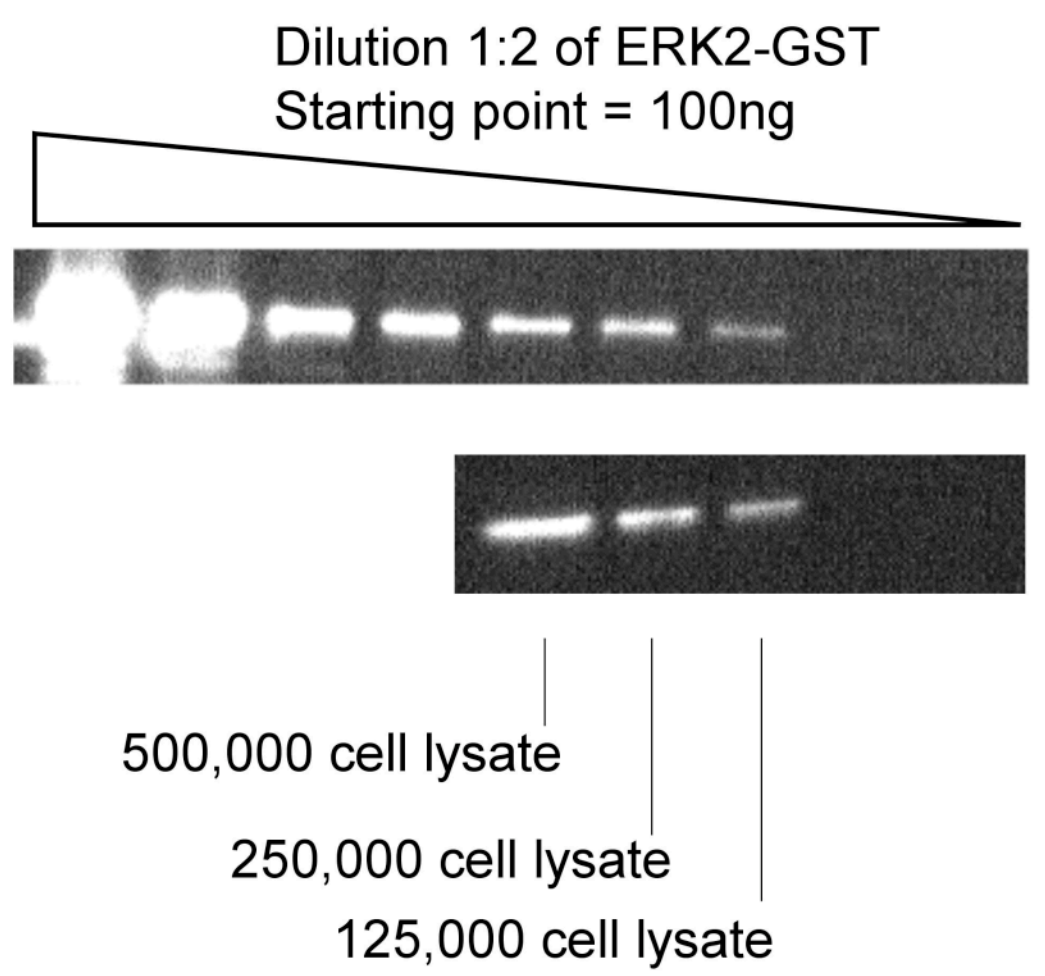
Dilution 1:2 of ERK2-GST
Starting point = 100ng
500,000 cell lysate
250,000 cell lysate
125,000 cell lysate

2B

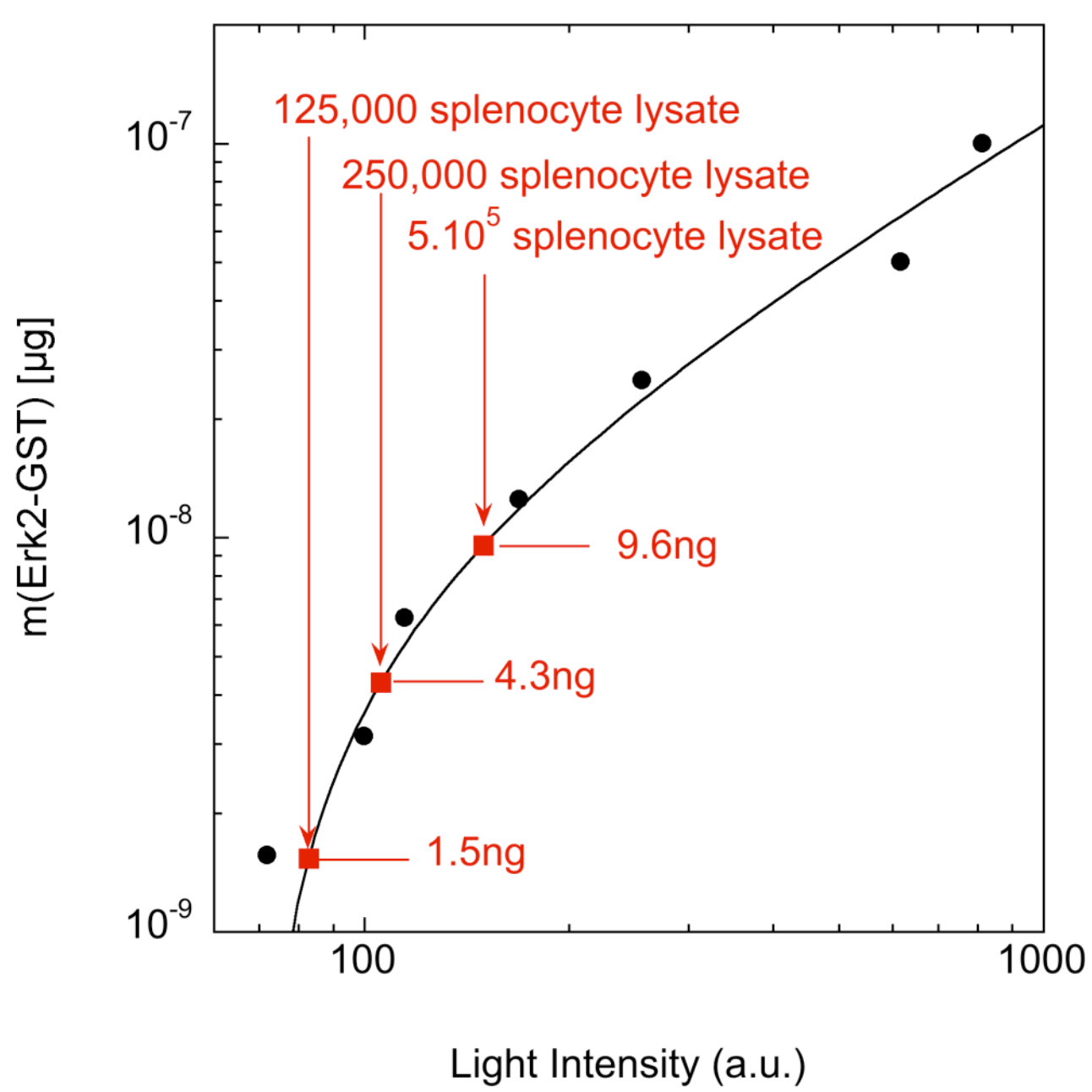
125,000 splenocyte lysate
250,000 splenocyte lysate
5.10^5 splenocyte lysate
9.6ng
4.3ng
1.5ng
m(Erk2-GST) [µg]
10^-7
10^-8
10^-9
100
1000
Light Intensity (a.u.)
