# Supplementary material for: Modeling T Cell Antigen Discrimination Based on Feedback Control of Digital ERK Responses
Source: PLoS Biol. 2005 Oct 25;3(11):e356. doi: 10.1371/journal.pbio.0030356 (PMC1262625; doi:10.1371/journal.pbio.0030356)
Supplement: Supplementary file 5 [file pbio.0030356.sg004.pdf]

A B C

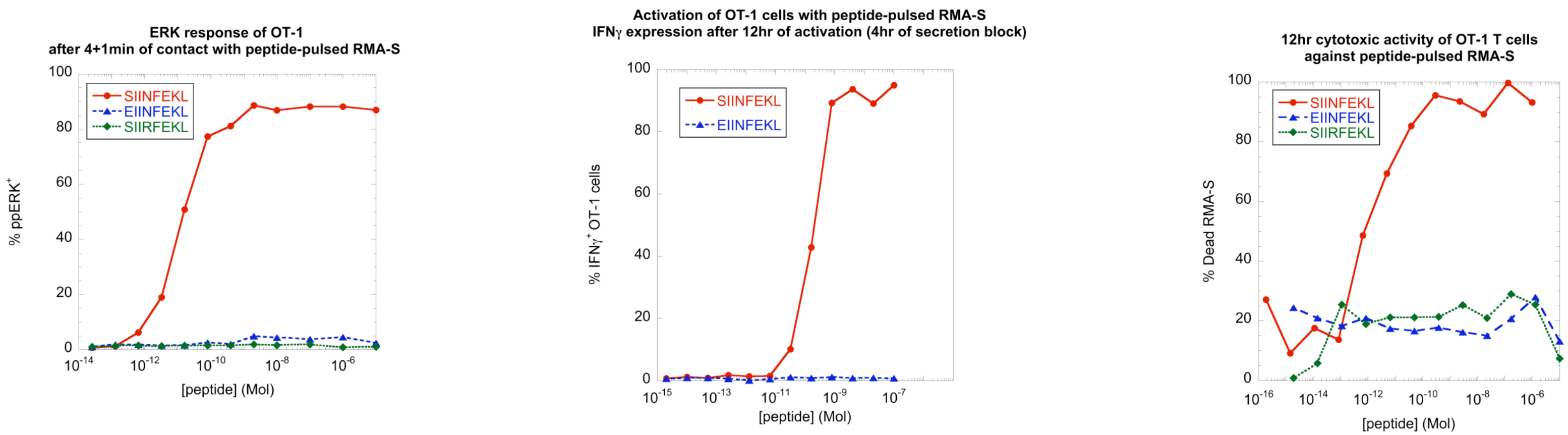

ERK response of OT-1
after 4+1min of contact with peptide-pulsed RMA-S
% ppERK+
[peptide] (Mol)
SIINFEKL
EIINFEKL
SIIRFEKL
Activation of OT-1 cells with peptide-pulsed RMA-S
IFNγ expression after 12hr of activation (4hr of secretion block)
% IFNγ+ OT-1 cells
[peptide] (Mol)
SIINFEKL
EIINFEKL
12hr cytotoxic activity of OT-1 T cells
against peptide-pulsed RMA-S
% Dead RMA-S
[peptide] (Mol)
SIINFEKL
EIINFEKL
SIIRFEKL
