# Supplementary material for: Modeling T Cell Antigen Discrimination Based on Feedback Control of Digital ERK Responses
Source: PLoS Biol. 2005 Oct 25;3(11):e356. doi: 10.1371/journal.pbio.0030356 (PMC1262625; doi:10.1371/journal.pbio.0030356)
Supplement: Supplementary file 6 [file pbio.0030356.sg005.pdf]

S5

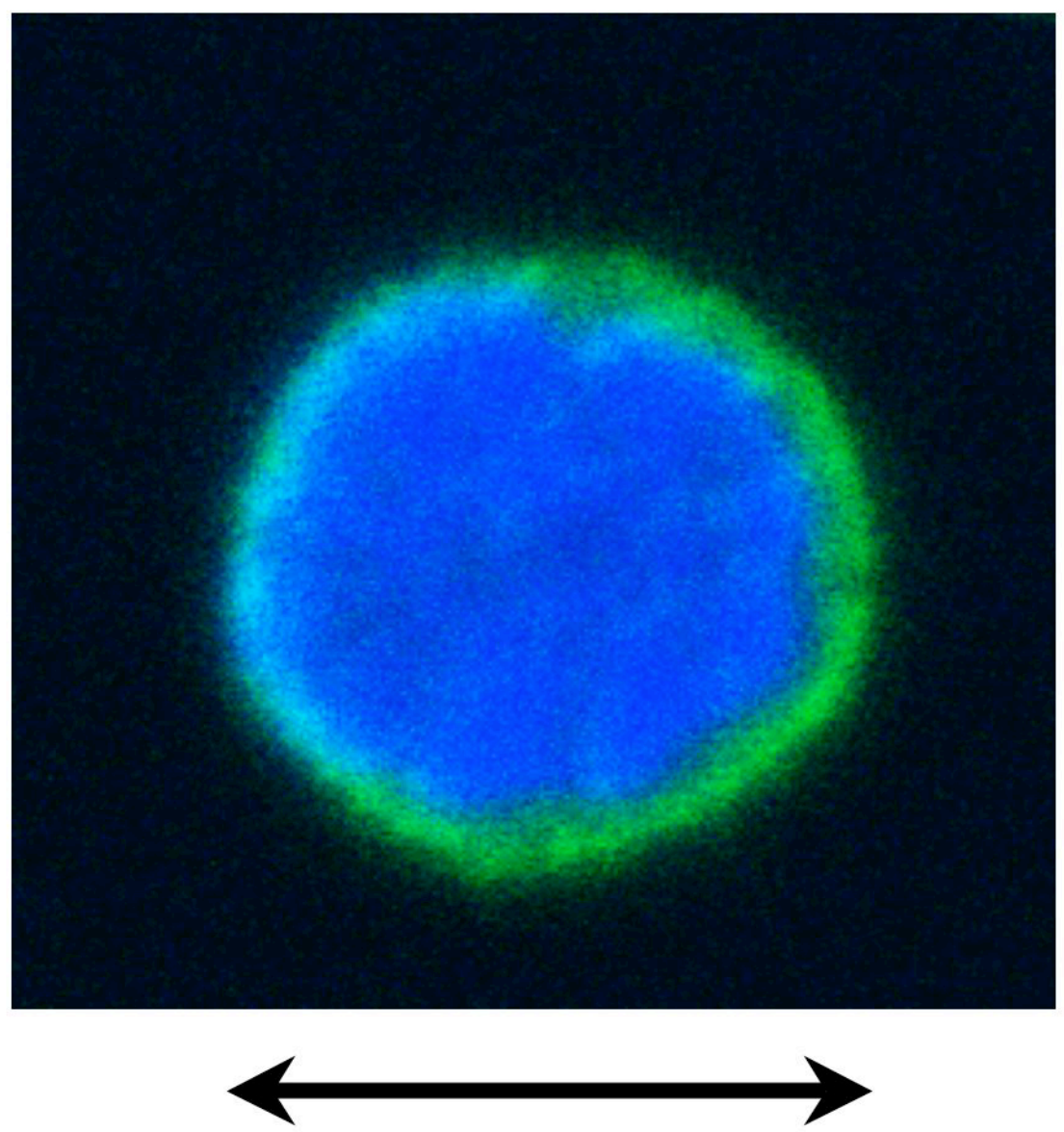

5.6μm

Confocal microscopy
after intracellular immunostaining
Green: ZAP70 (cytoplasm)
Blue: DAPI (nucleus)
