# Supplementary material for: Modeling T Cell Antigen Discrimination Based on Feedback Control of Digital ERK Responses
Source: PLoS Biol. 2005 Oct 25;3(11):e356. doi: 10.1371/journal.pbio.0030356 (PMC1262625; doi:10.1371/journal.pbio.0030356)
Supplement: Supplementary file 7 [file pbio.0030356.sg006.pdf]

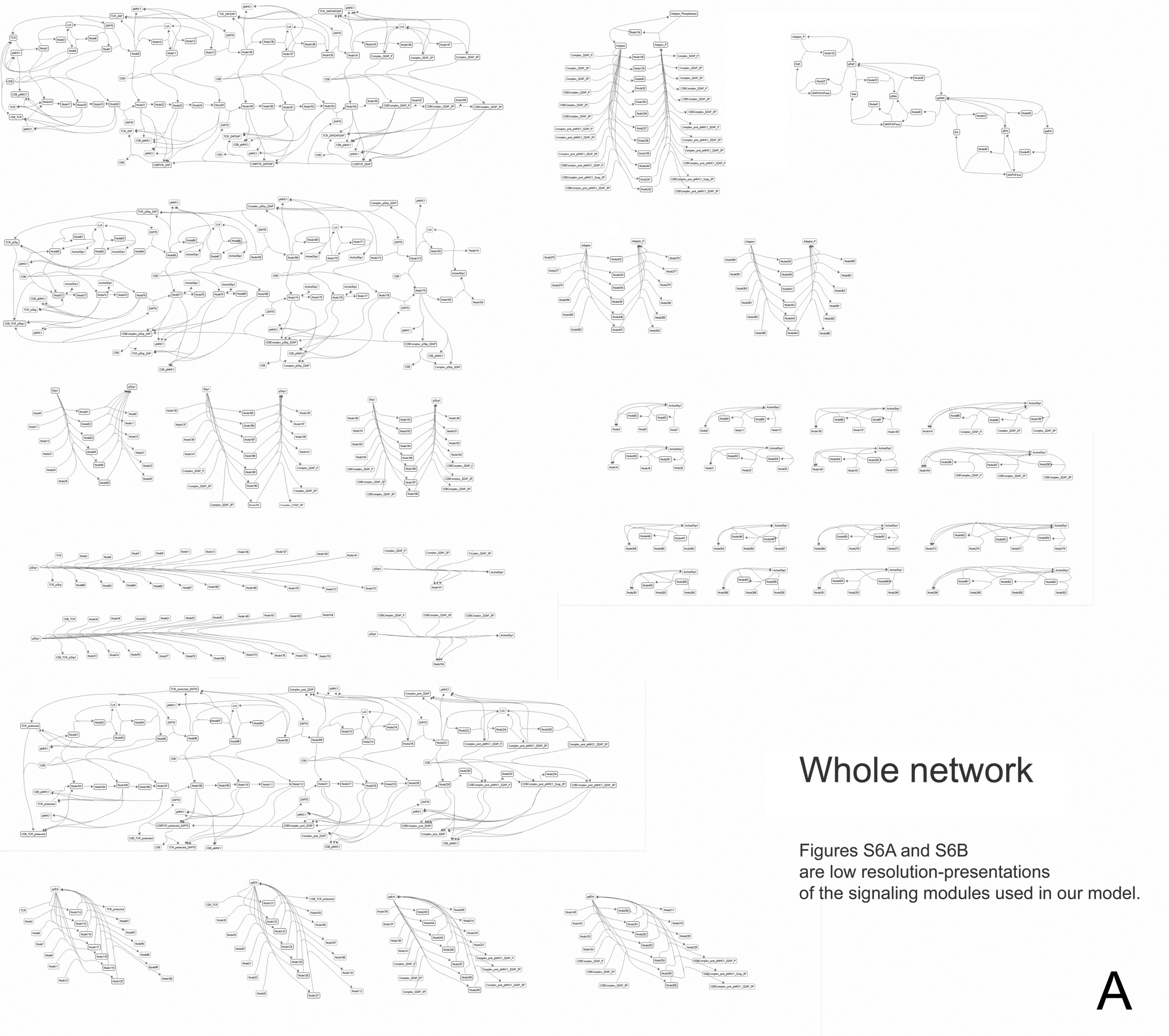

Whole network

Figures S6A and S6B are low resolution-presentations of the signaling modules used in our model.

A

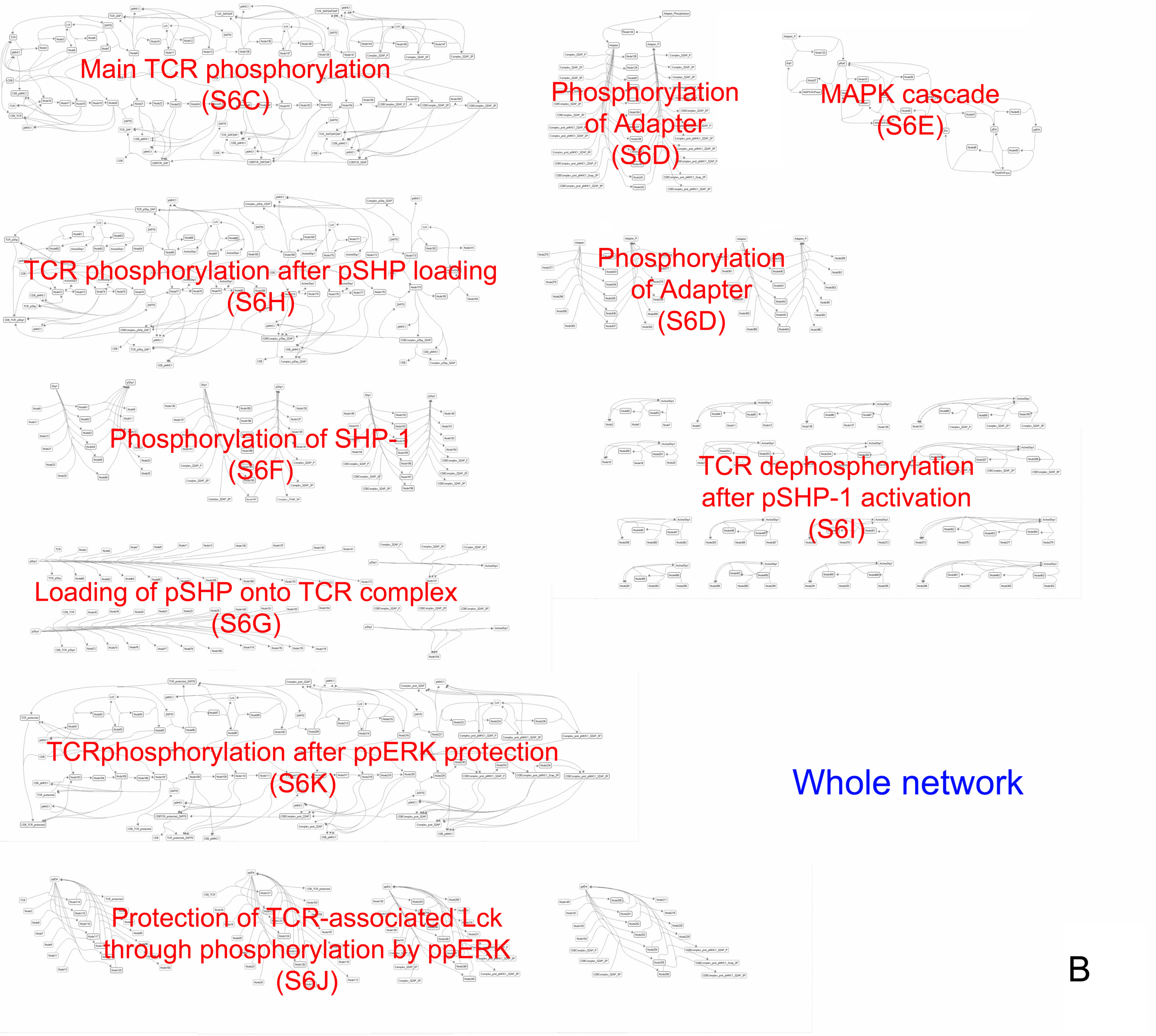
Main TCR phosphorylation (S6C)
Phosphorylation of Adapter (S6D)
MAPK cascade (S6E)
TCR phosphorylation after pSHP loading (S6H)
Phosphorylation of Adapter (S6D)
Phosphorylation of SHP-1 (S6F)
TCR dephosphorylation after pSHP-1 activation (S6I)
Loading of pSHP onto TCR complex (S6G)
TCRphosphorylation after ppERK protection (S6K)
Whole network
Protection of TCR-associated Lck through phosphorylation by ppERK (S6J)
B

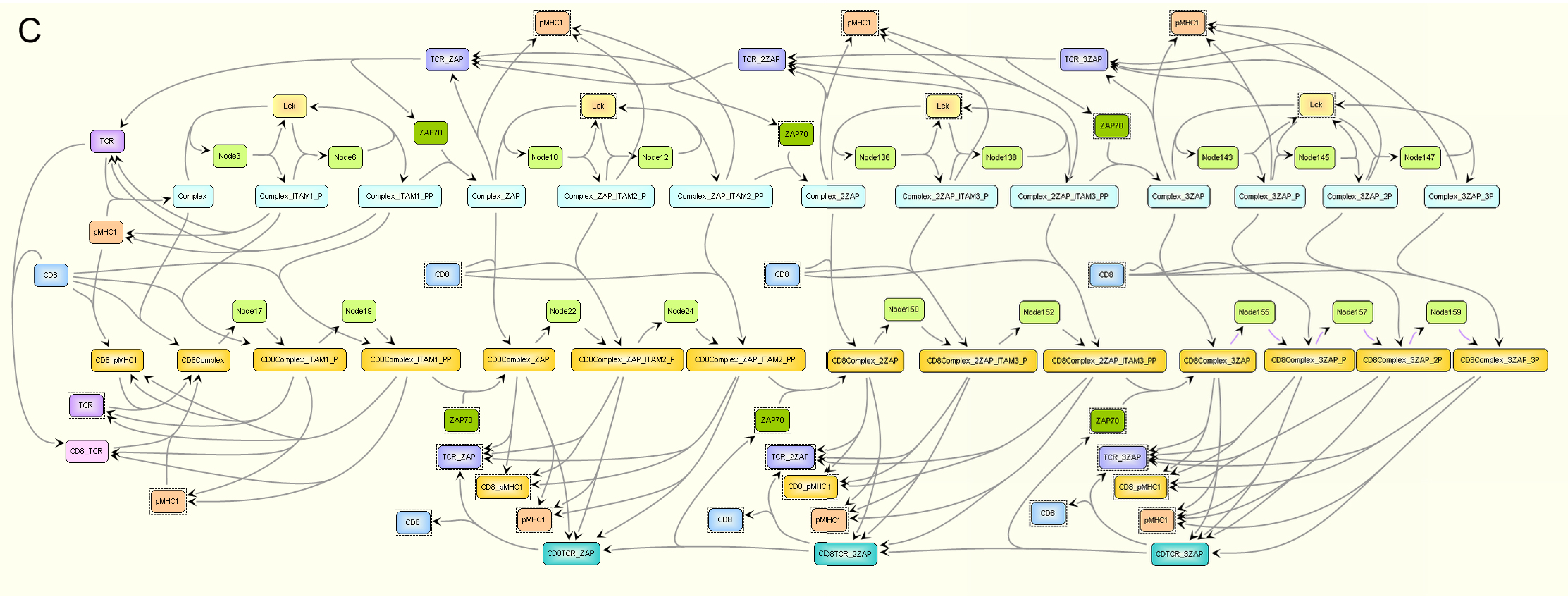

Main TCR network

D

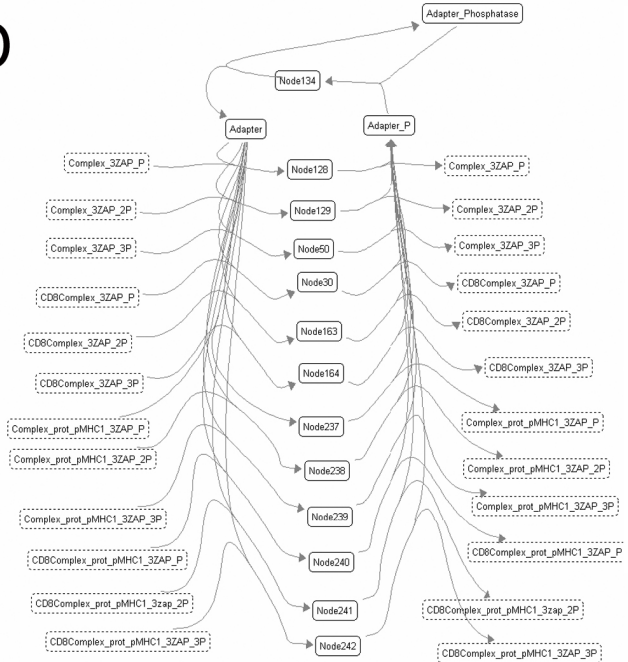

Phosphorylation of Adapter

E

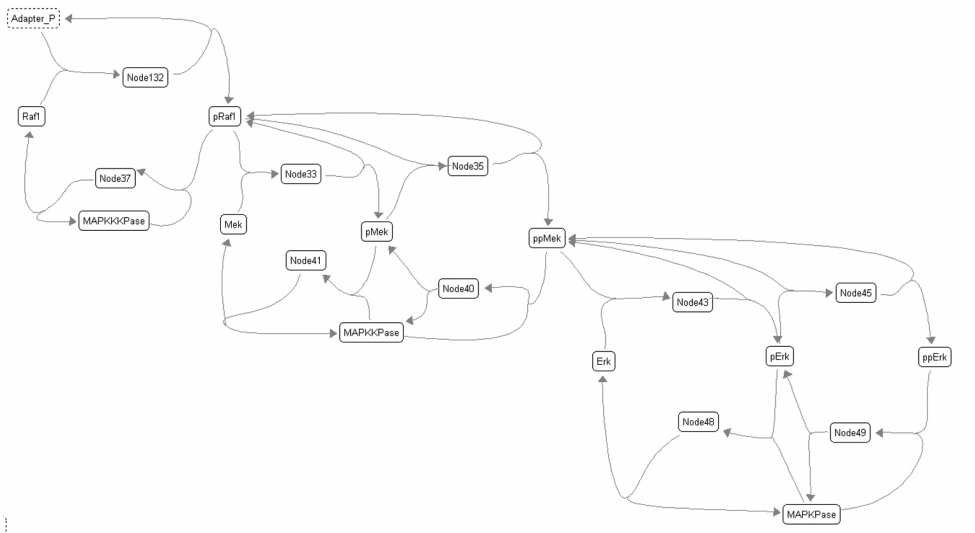

MAPK cascade

F

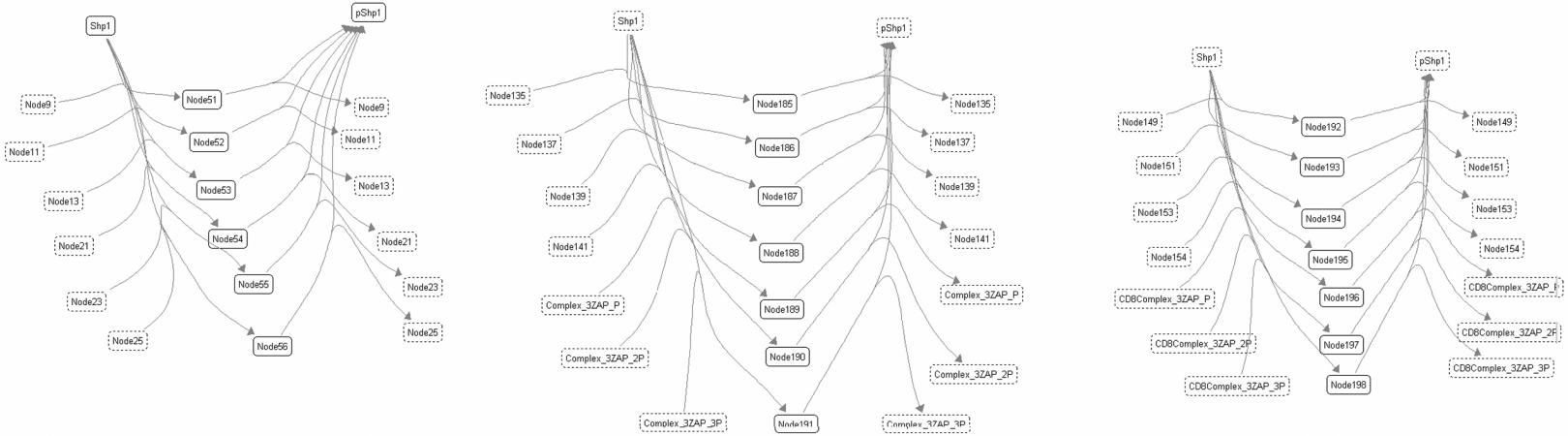

Phosphorylation of SHP-1

G

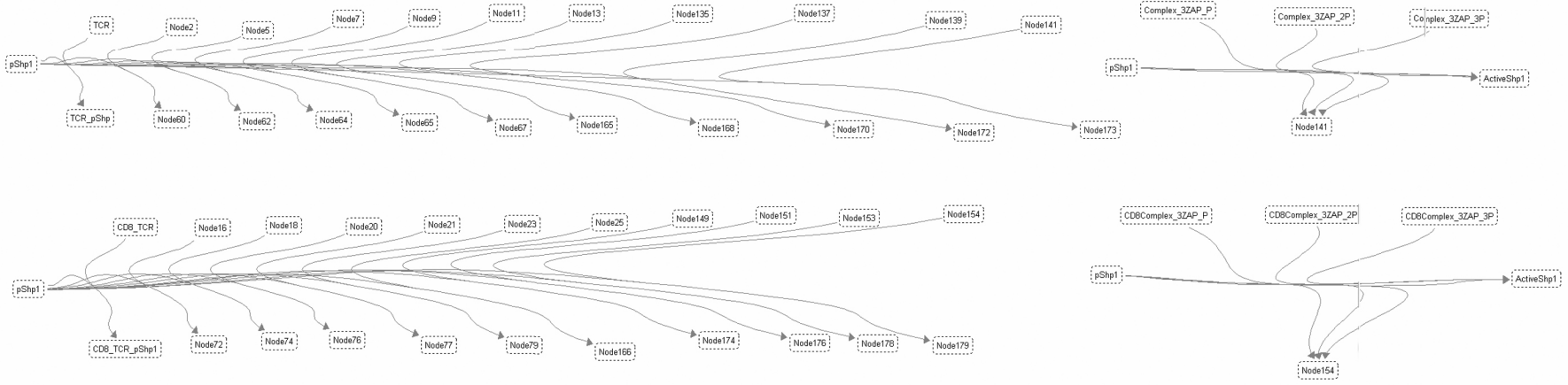

Loading of pSHP onto TCR complex

H

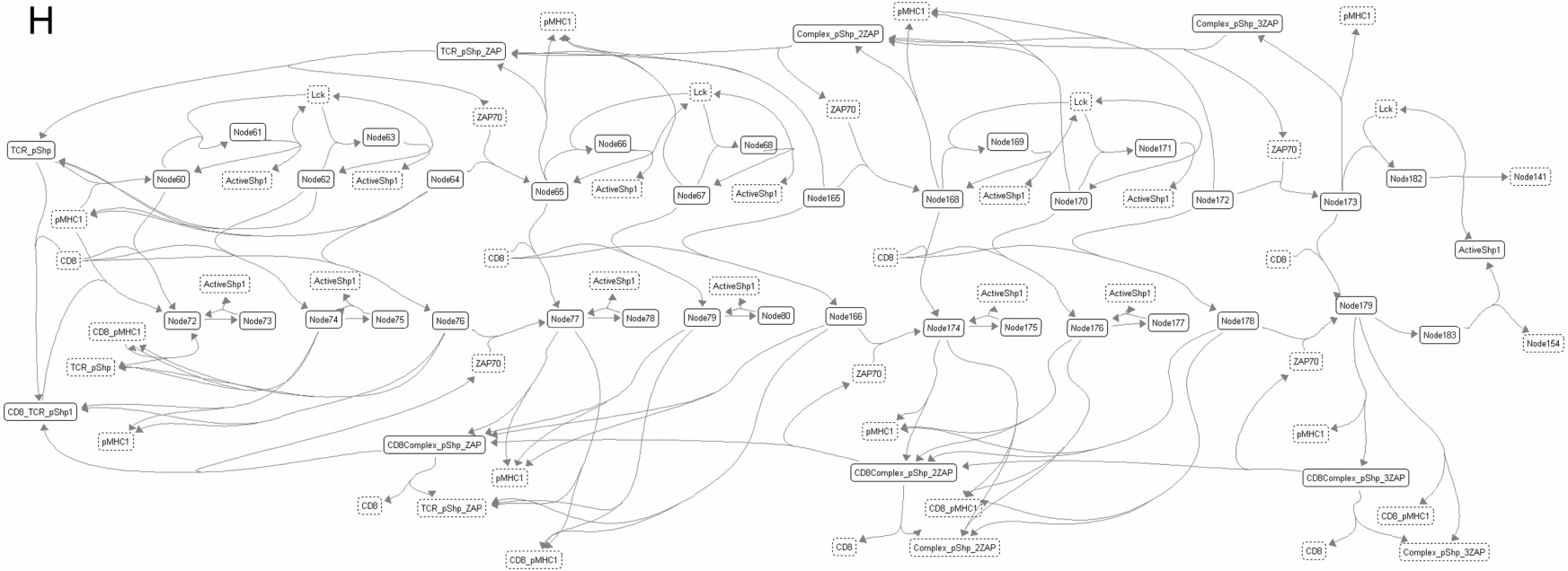

TCR phosphorylation after pSHP loading

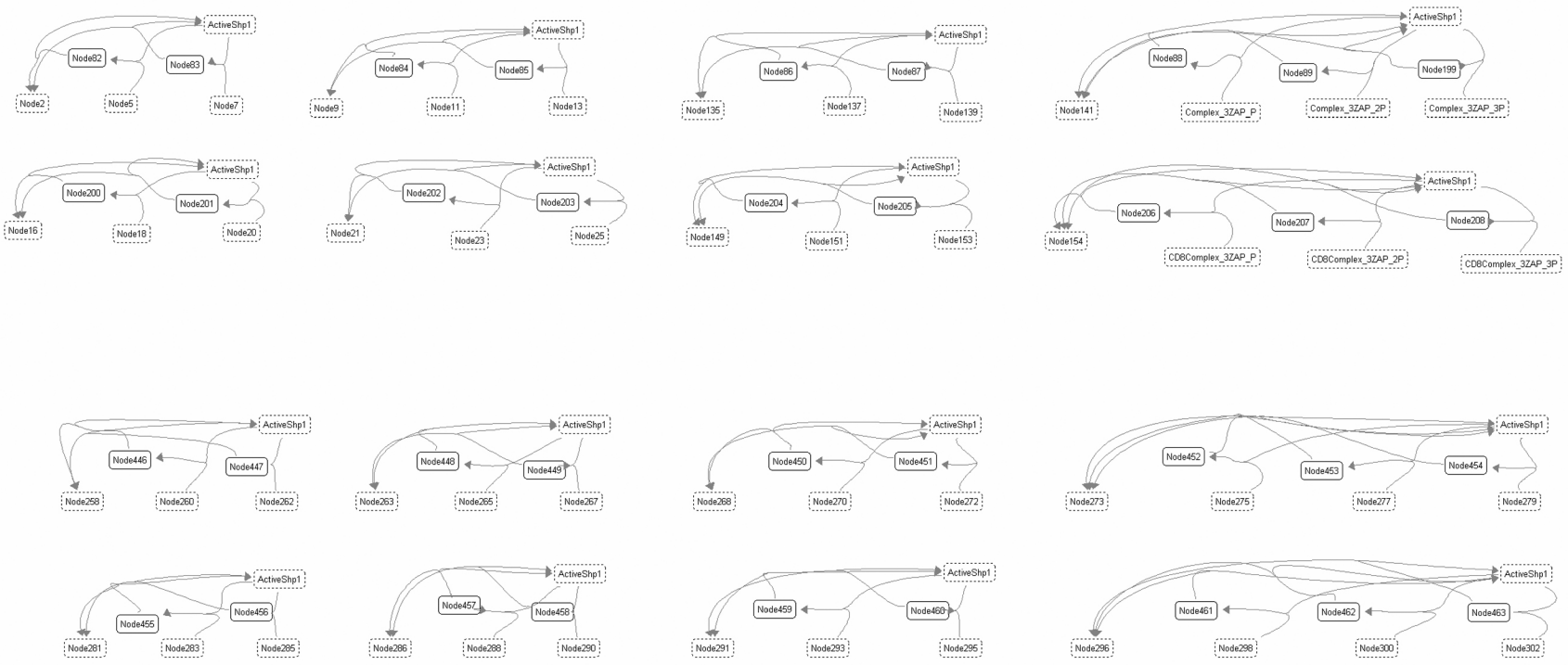

TCR dephosphorylation by activated SHP-1

J

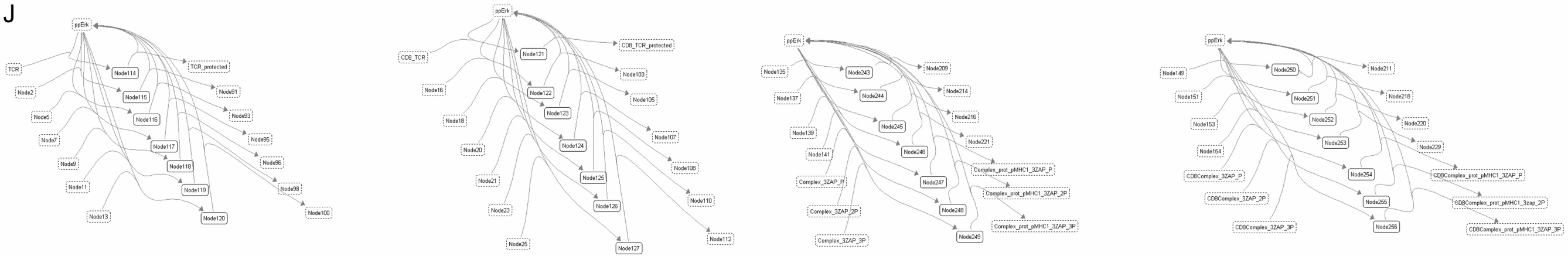

Protection of TCR-associated Lck through phosphorylation by ppERK

K

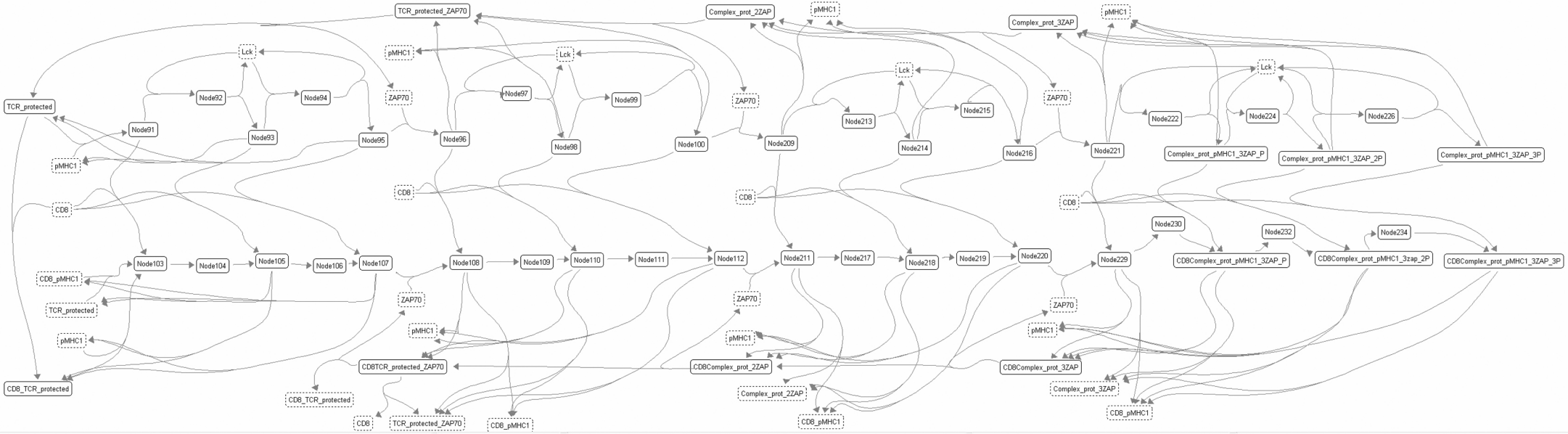

TCR phosphorylation after ppERK protection
