# Supplementary material for: Modeling T Cell Antigen Discrimination Based on Feedback Control of Digital ERK Responses
Source: PLoS Biol. 2005 Oct 25;3(11):e356. doi: 10.1371/journal.pbio.0030356 (PMC1262625; doi:10.1371/journal.pbio.0030356)
Supplement: Supplementary file 8 [file pbio.0030356.sg007.pdf]

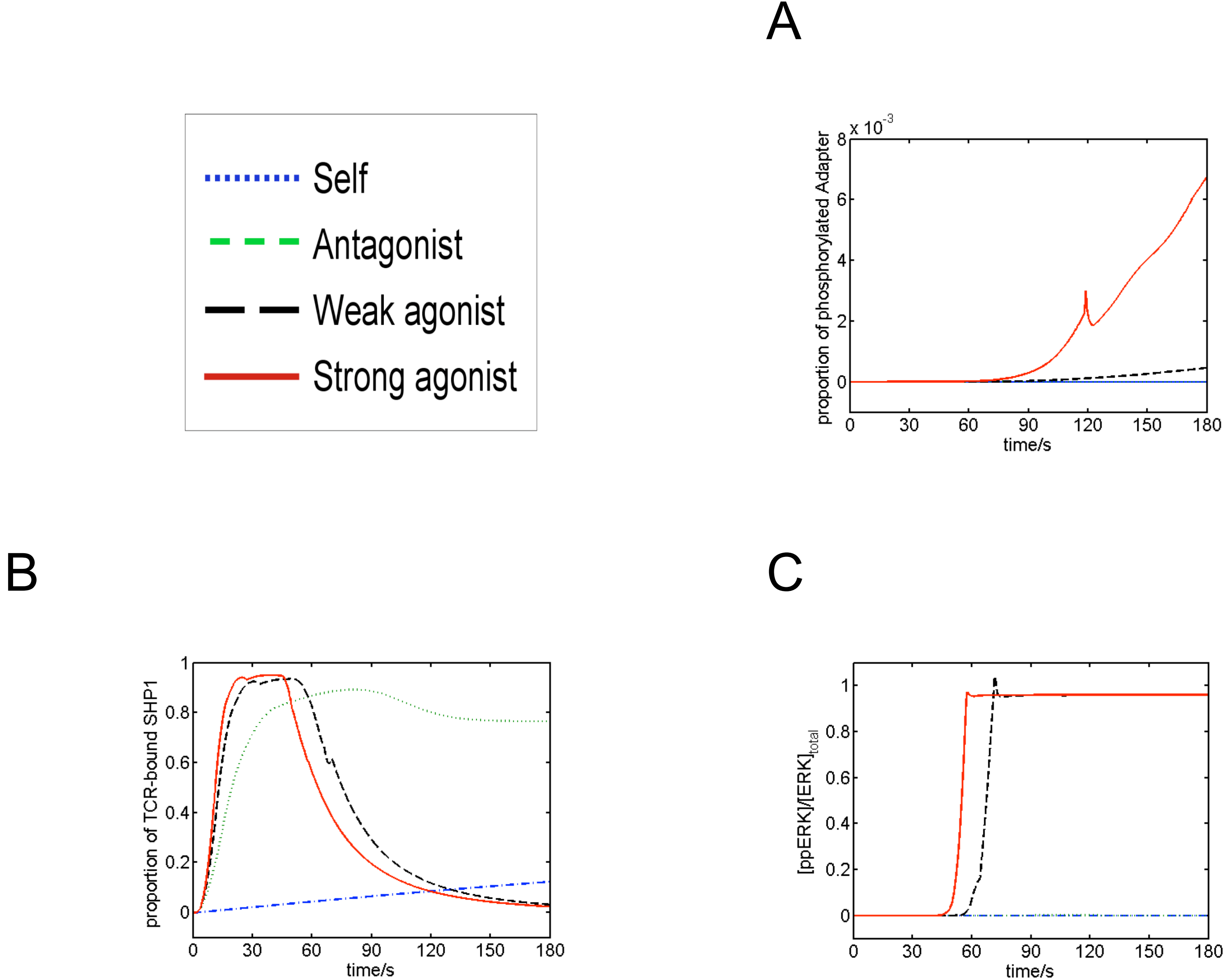

Self
Antagonist
Weak agonist
Strong agonist
A
x 10^-3
8
6
4
2
0
proportion of phosphorylated Adapter
0 30 60 90 120 150 180
time/s
B
1
0.8
0.6
0.4
0.2
0
proportion of TCR-bound SHP1
0 30 60 90 120 150 180
time/s
C
1
0.8
0.6
0.4
0.2
0
[ppERK]/[ERK]total
0 30 60 90 120 150 180
time/s
