# Supplementary material for: Modeling T Cell Antigen Discrimination Based on Feedback Control of Digital ERK Responses
Source: PLoS Biol. 2005 Oct 25;3(11):e356. doi: 10.1371/journal.pbio.0030356 (PMC1262625; doi:10.1371/journal.pbio.0030356)
Supplement: Supplementary file 9 [file pbio.0030356.sg008.pdf]

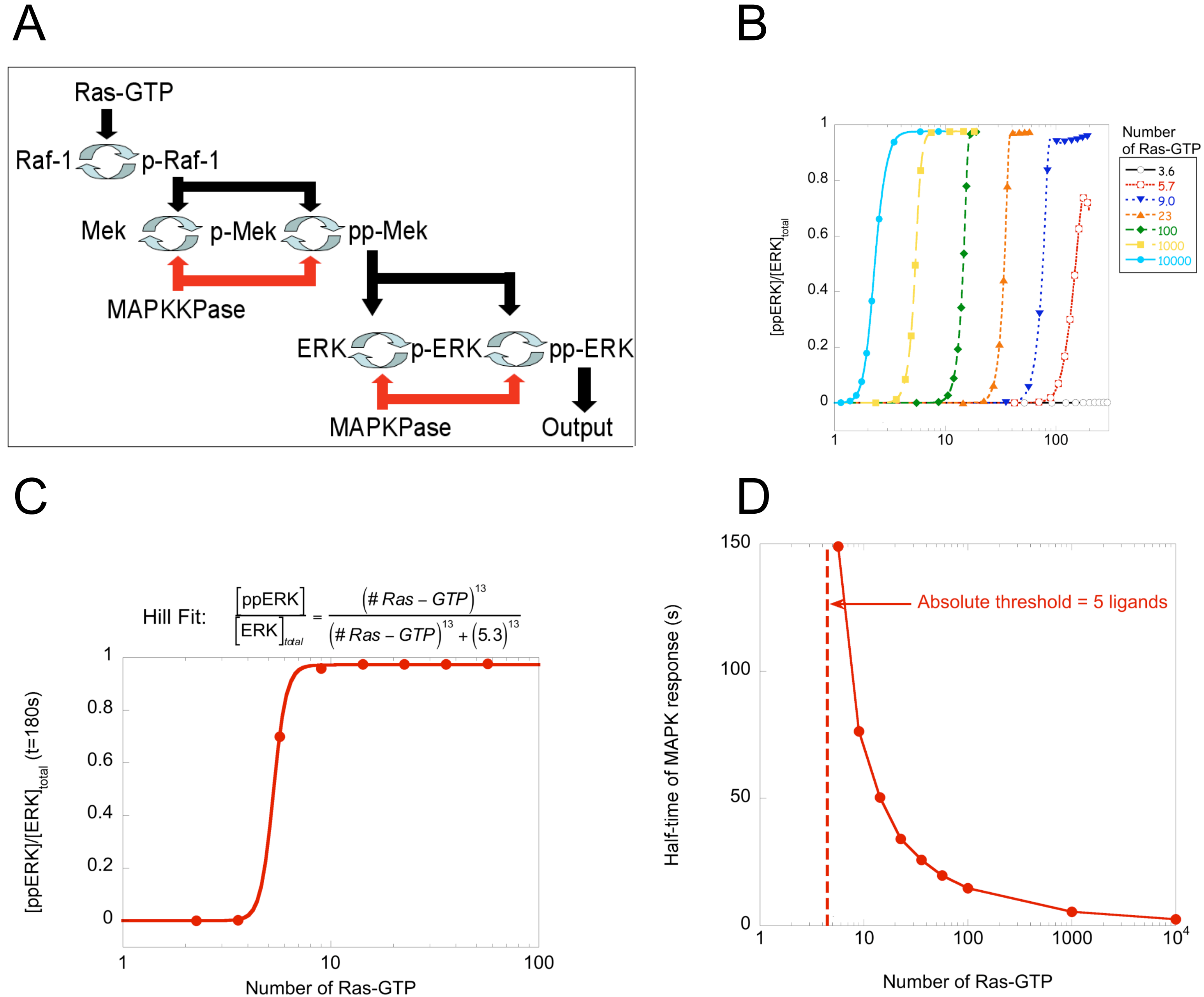
A
Ras-GTP
Raf-1
p-Raf-1
Mek
p-Mek
pp-Mek
MAPKKPase
ERK
p-ERK
pp-ERK
MAPKPase
Output
B
[ppERK]/[ERK]total
Number of Ras-GTP
3.6
5.7
9.0
23
100
1000
10000
C
Hill Fit: $\frac{[ppERK]}{[ERK]_{total}} = \frac{(\#Ras-GTP)^{13}}{(\#Ras-GTP)^{13}+(5.3)^{13}}$
[ppERK]/[ERK]total (t=180s)
Number of Ras-GTP
D
Half-time of MAPK response (s)
Absolute threshold = 5 ligands
Number of Ras-GTP
