# Supplementary material for: Modeling T Cell Antigen Discrimination Based on Feedback Control of Digital ERK Responses
Source: PLoS Biol. 2005 Oct 25;3(11):e356. doi: 10.1371/journal.pbio.0030356 (PMC1262625; doi:10.1371/journal.pbio.0030356)
Supplement: Supplementary file 10 [file pbio.0030356.sg009.pdf]

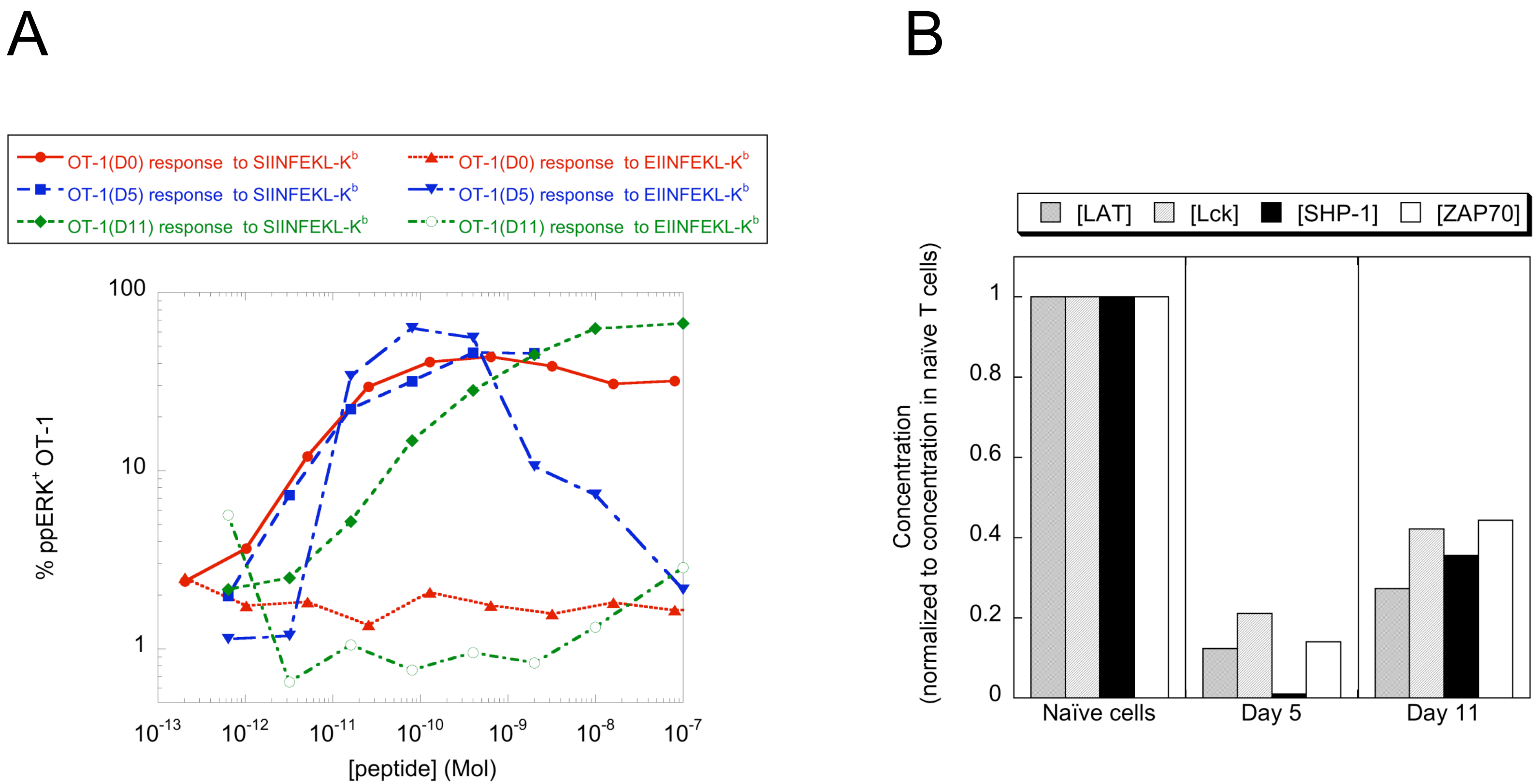
A
OT-1(D0) response to SIINFEKL-K^b
OT-1(D5) response to SIINFEKL-K^b
OT-1(D11) response to SIINFEKL-K^b
OT-1(D0) response to EIINFEKL-K^b
OT-1(D5) response to EIINFEKL-K^b
OT-1(D11) response to EIINFEKL-K^b
% ppERK+ OT-1
100
10
1
10^-13 10^-12 10^-11 10^-10 10^-9 10^-8 10^-7
[peptide] (Mol)
B
[LAT] [Lck] [SHP-1] [ZAP70]
Concentration
(normalized to concentration in naïve T cells)
1
0.8
0.6
0.4
0.2
0
Naïve cells
Day 5
Day 11
