# Supplementary material for: Modeling T Cell Antigen Discrimination Based on Feedback Control of Digital ERK Responses
Source: PLoS Biol. 2005 Oct 25;3(11):e356. doi: 10.1371/journal.pbio.0030356 (PMC1262625; doi:10.1371/journal.pbio.0030356)
Supplement: Supplementary file 11 [file pbio.0030356.sg010.pdf]

A

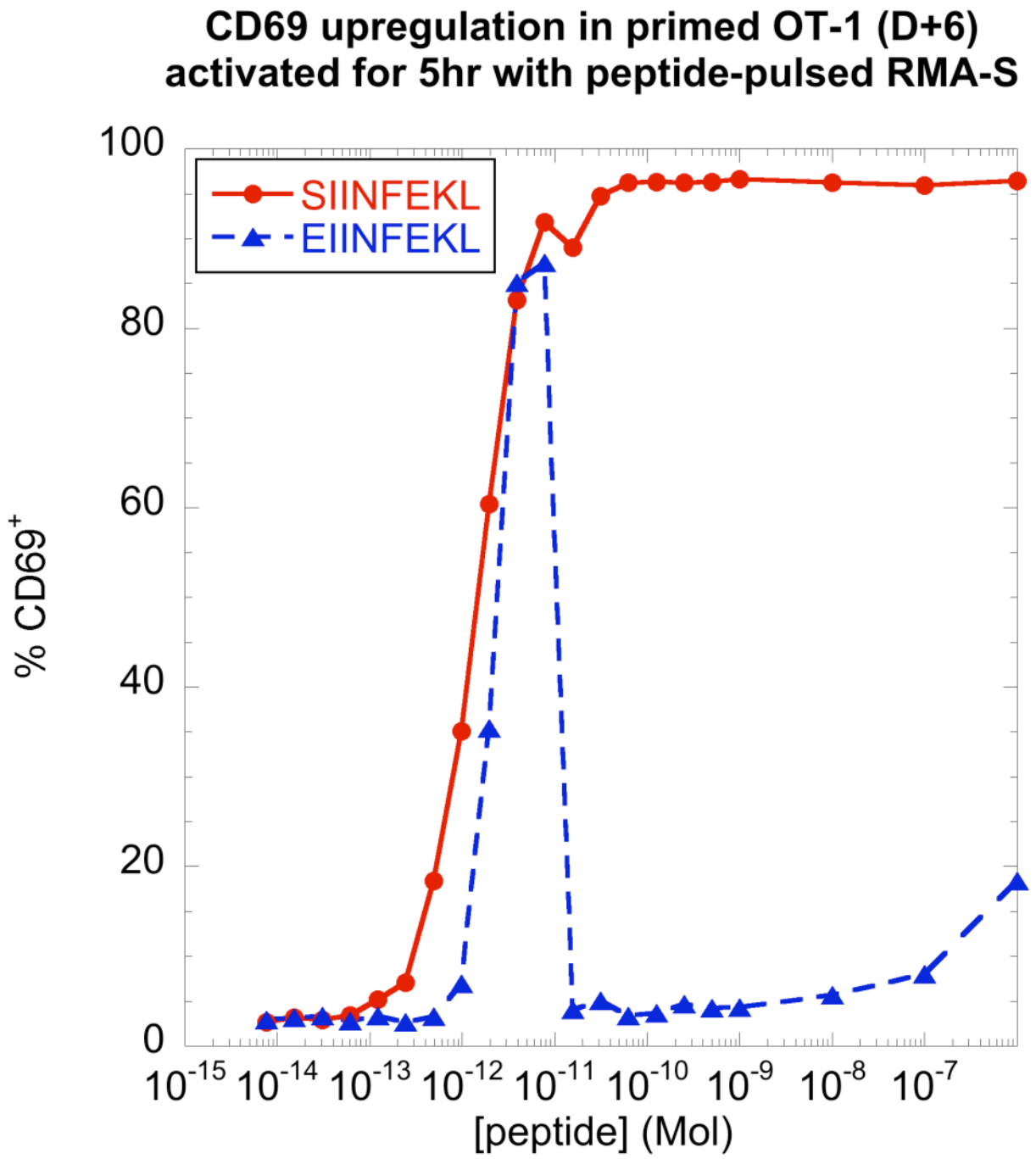
CD69 upregulation in primed OT-1 (D+6)
activated for 5hr with peptide-pulsed RMA-S
SIINFEKL
EIINFEKL
% CD69+
100
80
60
40
20
0
10-15 10-14 10-13 10-12 10-11 10-10 10-9 10-8 10-7
[peptide] (Mol)

B

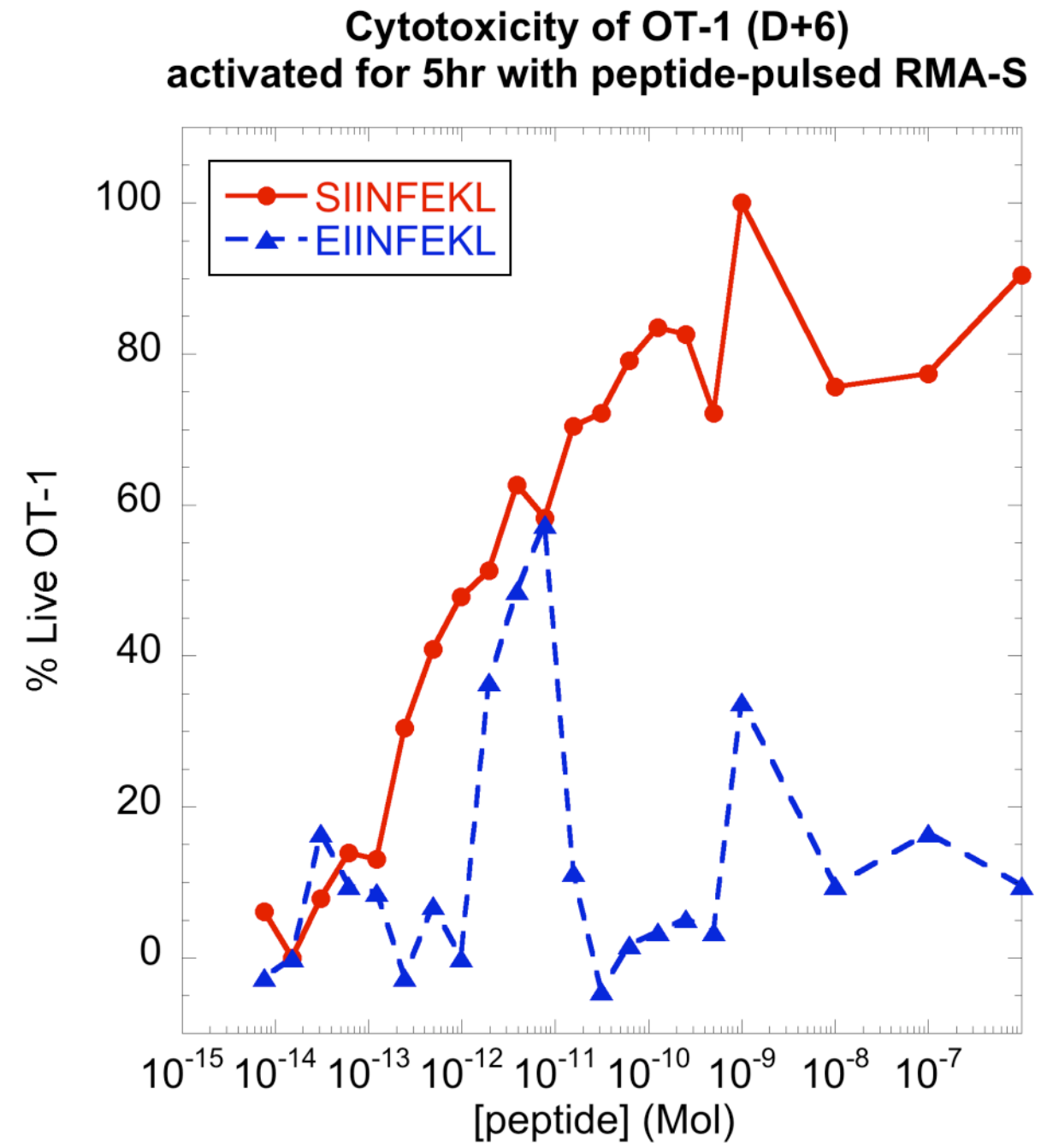
Cytotoxicity of OT-1 (D+6)
activated for 5hr with peptide-pulsed RMA-S
SIINFEKL
EIINFEKL
% Live OT-1
100
80
60
40
20
0
10-15 10-14 10-13 10-12 10-11 10-10 10-9 10-8 10-7
[peptide] (Mol)
