# Supplementary material for: Modeling T Cell Antigen Discrimination Based on Feedback Control of Digital ERK Responses
Source: PLoS Biol. 2005 Oct 25;3(11):e356. doi: 10.1371/journal.pbio.0030356 (PMC1262625; doi:10.1371/journal.pbio.0030356)
Supplement: Supplementary file 12 [file pbio.0030356.sd002.doc]

**Protocol S2.**

Experiments show that individual T cells exhibit a digital (all-or-none) ppERK response above a threshold *x_0_* of pMHC presentation on antigen-presenting cells (APC). Thus, the probability of response *r(x, x_0_)* at the individual cell level is:

$$\left\{\begin{matrix} r(x,x_{0})=1 for x>x_{0} \\ r(x,x_{0})=0 for x<x_{0} \end{matrix}\right.$$

where *x_0_* is the ligand threshold of T cell ppERK response. Yet when we measure the percentage of ppERK-responding cells at the population level, we find a shallow dose response *R(y):*

$$R(y)=\frac{y^{n_{Hill}}}{y^{n_{Hill}}+y_{0}^{n_{Hill}}},$$

where *y_0_* is the mid-point and *n_Hill_* is the Hill coefficient measuring the steepness of the response. At the individual cell level, *n_Hill_* is infinite, while, at the population level, *n_Hill_* is typically 1.9 (Figure 2).

To reconcile these two observations, one must take into account the biological variation (“noise”) in the level of pMHC presentation among the APC in a population as well as the variation in sensitivity of the ppERK response among the responding T cells to convert an individual dose response into a population dose response.

The distribution of presentation of K^b^/SIINFEKL on the surface of antigen-presenting cells is log-normal (Supplementary Figure 1). The probability *p(x,Y)* to present *x* ligands when *Y* ligands are presented on average is:

$$p(x,Y)=\frac{1}{x\sigma\sqrt{2\pi}}\exp\left(-\frac{\left(\ln x-\ln Y\right)^{2}}{2\sigma^{2}}\right),$$

where *σ* is the standard deviation of the distribution of ligands. In the case of this lognormal distribution, the coefficient of variation is determined only by **

$$\sigma=\sqrt{\ln\left(1+\left(\frac{CV}{100}\right)^{2}\right)}.$$

A simple convolution of ligand distribution and individual dose response yields the population dose-response:

$$R(y)=\int_{0}^{\infty} dx p(x,y)r(x,x_{0})=\frac{1}{2}erfc\left(\frac{\ln x_{0}-\ln y}{\sigma\sqrt{2}}\right),$$

where $erfc(u)=\frac{2}{\sqrt{\pi}}\int_{0}^{\infty} dt\exp(-t^{2}).$This can be approximated as a Hill dose-response with a Hill coefficient of:

$$n_{Hill}=\frac{4}{\sigma\sqrt{2\pi}}=\frac{4}{\sqrt{2\pi\ln\left(1+\left(\frac{CV}{100}\right)^{2}\right)}}.$$

Typically RMA-S cells present K^b^/SIINFEKL with a coefficient of variation *CV_presentatio_*_n_ = 55+/-5% (Supplementary Figure 1). Thus, the noise in ligand presentation will reduce the infinite Hill coefficient (observed at the individual cell level) to *n_Hill_* = 3.1 at the population level. To account for the experimentally measured value for Hill coefficient of 1.9, one must assume noise in the threshold of T cell activation. Assuming a log-normal distribution of *x_0_* with a coefficient of variation of *CV_threshold_* = we can compute a population dose response:

$$R(y)=\int_{0}^{\infty} dx_{0}p(x_{0},y_{0})\int_{0}^{\infty} dx p(x,y)r(x,x_{0})=\frac{1}{2}\int_{0}^{\infty} dx_{0} p(x_{0},y_{0})erfc\left(\frac{\ln x_{0}-\ln y}{\sigma\sqrt{2}}\right),$$

A systematic plot of the resulting Hill coefficient for *R* as a function of *CV_threshold_* shows that a value of 75% for *CV_threshold_* for the noise in T cell activation threshold (combined with *CV_presentation_* = 55% for ligand presentation) yields *n_Hill_* = 1.9 for T cell responses at the population level.

Hence, the shallow dose-response for ERK phosphorylation at the population level can be reconciled with the digital response documented by flow cytometry at the individual cell level (and obtained in the computer simulation), by taking into account the experimentally measured noise in ligand presentation and assuming a log-normally-distributed noise in the activation threshold of individual cells.
