# Supplementary material for: Modeling T Cell Antigen Discrimination Based on Feedback Control of Digital ERK Responses
Source: PLoS Biol. 2005 Oct 25;3(11):e356. doi: 10.1371/journal.pbio.0030356 (PMC1262625; doi:10.1371/journal.pbio.0030356)
Supplement: Supplementary file 13 [file pbio.0030356.sd003.doc]

**Protocol S3.**

**1) TCR-pMHC association/dissociation:**

The interaction of pMHC with TCR has been well studied using surface plasmon resonance (SPR) to measure the interaction of soluble versions of these membrane-associated proteins, one of which is immobilized on the analysis chip [1]. Typically, these studies show that:

$$\begin{matrix} \left(k_{association}\left(\text{pMHC-TCR}\right)\right)_{SPR}=10^{4} Mol^{-1}.s^{-1}, \\ \left(k_{dissociation}\left(\text{pMHC-TCR}\right)\right)_{SPR}=0.05 \text{to} 1 s^{-1}. \end{matrix}$$

The slow on-rate implies a substantial activation barrier for pMHC-TCR interaction in solution. We needed to convert this SPR number to estimate the association rate of pMHC and TCR when attached to the surface of an antigen presenting cell (APC) and a T cell. The diffusion limit for pMHC and TCR association in SPR experiments (*i.e., in* three dimensions) is:

*k_3D_*(diffusion) = 4 *N_a_* D_3D_(pMHC) *l*_pMHC-TCR_ = 5 x 10^7^ Mol^-1^.s^-1^ [2], where *N_a_* is the Avogadro Number, D_3D_(pMHC) is the coefficient of diffusion of pMHC in solution (typically*,* D_3D_(pMHC) = 10^-9^ dm^2^/s; the TCR does not diffuse in SPR experiments because it is anchored onto the chip) and *l*_pMHC-TCR_ is the typical scale of the pMHC-TCR interaction area (*l*_pMHC-TCR_ = 5 x 10^-9^ dm). The probability of successful association (*p_association_*) between pMHC and TCR is then:

*p_association_*= *k_SPR_*(pMHC-TCR)/ *k_3D_*(diffusion) = 2 x 10^-4^

for these unconstrained conditions. The frequency of successful pMHC-TCR interaction is thus very low, which poses a challenge for understanding the speed of T cell activation upon T cell interaction with an APC. On the other hand, all pMHC-TCR interactions happen at the T:APC interface such that these surface proteins are confined spatially and electrostatically. Consequently, we increased the probability of pMHC-TCR association on membrane by a factor of 7.4 in our simulation, and set:

*p_association_=*1.5 x 10^-3^*.*

Because of membrane localization, we had to convert association rates in two dimensions (2D), into association rates in three dimensions. Essentially, for a surface *S* and a cytoplasmic volume *V*, we needed to match the 2D and 3D frequencies of successful pMHC-TCR association:

$$\begin{matrix} p_{success}=\frac{k_{2D}}{S}\times N_{pMHC}N_{TCR}=\frac{k_{3D}}{V}\times N_{pMHC}N_{TCR}, \\ \Rightarrow k_{3D}=k_{2D}\times\left(\frac{V}{S}\right)=k_{2D}(diffusion)\times p_{association}\times\left(\frac{V}{S}\right). \end{matrix}$$

Classical estimates yield the association rates for diffusion-limited chemical interactions in 2D (typically for a membrane receptor on the surface of T cells, D_2D_ = 0.1 *μm^2^/s*):

$$k_{2D}(diffusion)=\frac{2\pi D_{2D}}{\ln\left(\frac{\sqrt{S/4\pi}}{r}\right)}\simeq8.10^{-2}\mu m^{2}/s.$$

For the naïve T cells under consideration, the diameter of the cells is 5.6 μm and the cytoplasmic volume is 15 fl (see Supplementary material S4), hence *V/S* = 0.15 μm (this is the typical thickness of the cytoplasm in naïve T cells). The final association rate used in our simulation is then:

$$\begin{matrix} k_{association}\text{(pMHC-TCR)=8.10}^{\text{-2}}\times0.15\times1.5\times10^{-3}=1.8\times10^{-5}\mu m^{3}.s^{-1} \\ \Rightarrow k_{association}\text{(pMHC-TCR)}=10^{4}Mol^{-1}.s^{-1} \end{matrix}$$

To give a more intuitive understanding of this number, given the density of TCR on the surface of naïve T cells (30,000 TCR on a 5.6 μm-diameter cell with a 15 fl cytoplasmic volume), we can estimate the frequency *f* of successful hit of a pMHC:

$$f=k_{association}[TCR]=10^{4}\times\frac{30000}{6.10^{23}\times15.10^{-15}}\simeq3.10^{-2} s^{-1}=\left(\frac{1}{30 s}\right).$$

This realistic hit frequency (derived from experimentally-measured association rates) is challenging, as we measured a typical time for ERK phosphorylation or for a calcium response to be 1 min. This emphasizes the need for fast and robust signal amplification downstream of pMHC-TCR engagement. The final values used for the simulation of the TCR signaling in naïve T cells are:

*k_association_*(pMHC-TCR) = 1 x 10^4^ Mol^-1^.s^-1^,

*k_dissociation_*(pMHC-TCR) = 0.05 to 1 s^-1^,

where the dissociation rates vary depending on the quality of the pMHC {typically for an agonist, *k_dissociation_*(pMHC-TCR) = 0.055 s^-1^ = (18 s)^-1^ while for the best non-agonist, *k_dissociation_*(pMHC-TCR) = 0.33 s^-1^ = (3 s)^-1^}.

**2) CD8-pMHC association/dissociation:**

The association and dissociation rates for human CD8αα and pMHC have been estimated at 37^0^C by surface plasmon resonance [3]:

$$\begin{matrix} \left(k_{association}\left(\text{CD8}\alpha\alpha\text{-pMHC}\right)\right)_{SPR}>10^{5} Mol^{-1}.s^{-1}, \\ \left(k_{dissociation}\left(\text{CD8}\alpha\alpha\text{-pMHC}\right)\right)_{SPR}>20 s^{-1}. \end{matrix}$$

The association equilibrium constant was estimated to be ~200 Mol. Thus, CD8αα and pMHC association is fast and transient. Following Wyer *et al.*’s unpublished data [3], the kinetics of association/dissociation of mouse CD8αα or CD8αβ with pMHC are similar to the kinetics of association/dissociation of human CD8αα with pMHC. Consequently, using the same 2D-3D conversion presented above for pMHC-TCR association, we used in our computer simulation:

*k_association_*(CD8-pMHC) = 3 x 10^5^ Mol^-1^.s^-1^,

*k_dissociation_*(CD8-pMHC) = 100 s^-1^.

**3) TCR phosphorylation:**

Several groups [4,5,6] have reported a precise order of phosphorylation of the 6 tyrosines present in the three Immunoreceptor Tyrosine-based Activation Motifs (ITAMs) of a monomer of TCR In our model, we took these measurements into account by implementing processivity in TCR phosphorylation. Each ITAM gets phosphorylated one by one, with a protection of the phospho-tyrosine doublet within each ITAM through ZAP70 binding. We also model the coupling of the phosphorylation of TCR to the downstream signaling cascade by implementing the conformational change in TCR documented by Aivazian & Stern [7]: ZAP70 phosphorylation and coupling with the adapter occurs only when all ITAMs’ have been phosphorylated and loaded with ZAP70. This processivity of TCR phosphorylation and coupling with downstream events constitute a realistic molecular implementation of the kinetic proofreading in our model of TCR signaling. Note that it is not as absolute a proofreading scheme as the ones used in other publications [8,9] because abrogated pMHC-TCR interactions here do not induce complete dephosphorylation of the TCR chains (this is realistic because ZAP70 is a tight binder of a fully-phosphorylated ITAM). Hence, the role of signaling feedbacks becomes crucial when only partial KP is performed (as in our realistic model).

**4) ZAP70 binding:**

Bunnell et al. [10] measured the turnover rate of ZAP70 in the contact area of an activated T cell using FRAP (Fluorescence Recovery After Photobleaching). At early times after initiation of signaling (t<2min), they report a turnover rate of 7.5s to 10s, which is dominated by the off-rate of ZAP70 binding to phospho-ITAMs. The dynamics of recruitment of ZAP70 is very fast. Therefore, we chose the diffusion limit for the association rate of ZAP70 and phospho-ITAM:

*k_association_* = 1.2 x 10^7^ Mol^-1^.s^-1^

*k_dissociation_* = 0.11 s^-1^

**5) Lck kinase activity:**

Ramer *et al.* [11] measured the catalytic activity of Lck using a synthetic peptide substrate. They measured a rate of 320 min^-1^ at room temperature. This relatively high catalytic turnover rate was used as such in our simulation. We set association rates and dissociation rates for a CD8-bound Lck onto at TCR substrate at (30 s^-1^).

**6) SHP-1 activation and phosphatase activity:**

The dynamics of SHP-1 activation is complex and difficult to document [12]. In our computer simulation we model the activation of SHP-1 as a two-step process: first, SHP-1 gets phosphorylated by an engaged TCR (this engagement implying activation of the TCR-bound Lck kinase), and the phospho-SHP-1 released into the cytoplasm; second, phospho-SHP-1 gets recruited to TCR containing Lck; further phosphorylation of the TCR-associated ZAP70 activates the phosphatase activity of SHP-1, which then dephosphorylates unprotected phospho-tyrosines (those of Lck, , and ZAP70 are the principal targets) followed by release of the phosphatase into the cytoplasm. Hence, in our model, SHP-1 works as an activatable, spreading negative regulator of TCR signaling, as described by Stefanova *et al.* [13]

Chiang & Sefton [14] reported that SHP-1 is a highly efficient phosphatase. At 7 nMol, SHP-1 could dephosphorylate Lck by 50% in 1 hr at 4°C. Hence, we assumed that SHP-1 binding to its substrate is tight, with a fast dephosphorylation activity at 37°C:

*k_associatio_*_n_ = 330,000 Mol^-1^.s^-1^,

*k*_dissociation_ = 0.05 s^-1^,

*k_cat_* = 5.4 s^-1^.

Note: for the simulation of the Day 5 phenotype (bell-shaped dose-response of ERK phosphorylation in OT-1 T cells activated with EIINFEKL-pulsed RMA-S APC and its correction by overexpression of SHP-1), we used faster kinetics for SHP-1 phosphorylation and loading onto TCR:

*k_associatio_*_n_ = 880,000 Mol^-1^.s^-1^,

*k*_dissociation_ = 0.05 s^-1^,

*k_cat_* = 5.4 s^-1^.

At present, we do not have any experimental justification for this increased strength in the SHP-1-associated negative feedback, which is the one kinetic parameter that must be adjusted as compared to naïve cells in the simulation data we present for Day5 cells for the simulation output to correctly predict experimental outcome.

**7) Adapter phosphorylation and ERK activation.**

To connect the phosphorylation of ZAP70 to the activation of the MAPK cascade, we modeled LAT, SLP76, and other adapters as a phenomenological single-step process. pZAP70 phosphorylates the adapter and allows it to activate Raf-1. This shortcut in the kinase cascade leading to ERK activation was implemented without loss of generality (our unpublished data), as it acts as a straightforward molecular transducer.

The cascade of kinases involved in ERK activation was simulated explicitly to account realistically for the signal amplification from pZAP70 to ppERK. We used the model proposed by Huang and Ferrell [15] with the following kinetic parameters for all enzymatic activities:

*k_associatio_*_n_ = 1.2 x 10^7^ Mol^-1^.s^-1^,

*k*_dissociation_ = 0.15 s^-1^,

*k_cat_* = 2.6 s^-1^.

We use such a large association rate (close to the diffusion limit for these enzymes) as a phenomenological attempt to deal with the presence of scaffolding in MAPK activation. These parameters yielded the fast, robust and digital response we observed experimentally.

**9) Lck protection.**

Upon ERK activation, TCR-associated Lck is serine-phosphorylated and protected from recruitment of SHP-1 and dephosphorylation/deactivation by this phosphatase [13]. The kinetics of this protection has not been documented but we reasoned that it must be fast to limit the effects of rapidly activated SHP-1 and show an effect on the phosphorylation status of TCR at early times [13]. Hence, we used:

*k_associatio_*_n_ = 1.0 x 10^7^ Mol^-1^.s^-1^,

*k*_dissociation_ = 2 s^-1^,

*k_cat_* = 3.2 s^-1^.

**References:**

1. Davis MM, Boniface JJ, Reich Z, Lyons D, Hampl J, et al. (1998) Ligand recognition by alpha beta T cell receptors. Annual Review of Immunology 16: 523-544.

2. Winter RB, Berg OG, von Hippel PH (1981) Diffusion-driven mechanisms of protein translocation on nucleic acids. 3. The Escherichia coli lac repressor--operator interaction: kinetic measurements and conclusions. Biochemistry 20: 6961-6977.

3. Wyer JR, Willcox BE, Gao GF, Gerth UC, Davis SJ, et al. (1999) T cell receptor and coreceptor CD8 alphaalpha bind peptide-MHC independently and with distinct kinetics. Immunity 10: 219-225.

4. Kersh EN, Shaw AS, Allen PM (1998) Fidelity of T cell activation through multistep T cell receptor zeta phosphorylation.[see comment]. Science 281: 572-575.

5. Pitcher LA, Young JA, Mathis MA, Wrage PC, Bartok B, et al. (2003) The formation and functions of the 21- and 23-kDa tyrosine-phosphorylated TCR zeta subunits. Immunological Reviews 191: 47-61.

6. Housden HR, Skipp PJ, Crump MP, Broadbridge RJ, Crabbe T, et al. (2003) Investigation of the kinetics and order of tyrosine phosphorylation in the T-cell receptor zeta chain by the protein tyrosine kinase Lck. European Journal of Biochemistry 270: 2369-2376.

7. Aivazian D, Stern LJ (2000) Phosphorylation of T cell receptor zeta is regulated by a lipid dependent folding transition. Nature Structural Biology 7: 1023-1026.

8. McKeithan TW (1995) Kinetic proofreading in T-cell receptor signal transduction. Proceedings of the National Academy of Sciences of the United States of America 92: 5042-5046.

9. Chan C, Stark J, George AJ (2004) Feedback control of T-cell receptor activation. Proceedings of the Royal Society of London - Series B: Biological Sciences 271: 931-939.

10. Bunnell SC, Hong DI, Kardon JR, Yamazaki T, McGlade CJ, et al. (2002) T cell receptor ligation induces the formation of dynamically regulated signaling assemblies. Journal of Cell Biology 158: 1263-1275.

11. Ramer SE, Winkler DG, Carrera A, Roberts TM, Walsh CT (1991) Purification and initial characterization of the lymphoid-cell protein-tyrosine kinase p56lck from a baculovirus expression system. Proceedings of the National Academy of Sciences of the United States of America 88: 6254-6258.

12. Plas DR, Johnson R, Pingel JT, Matthews RJ, Dalton M, et al. (1996) Direct regulation of ZAP-70 by SHP-1 in T cell antigen receptor signaling. Science 272: 1173-1176.

13. Stefanova I, Hemmer B, Vergelli M, Martin R, Biddison WE, et al. (2003) TCR ligand discrimination is enforced by competing ERK positive and SHP-1 negative feedback pathways.[see comment]. Nature Immunology 4: 248-254.

14. Chiang GG, Sefton BM (2001) Specific dephosphorylation of the Lck tyrosine protein kinase at Tyr-394 by the SHP-1 protein-tyrosine phosphatase. Journal of Biological Chemistry 276: 23173-23178.

15. Huang CY, Ferrell JE, Jr. (1996) Ultrasensitivity in the mitogen-activated protein kinase cascade. Proceedings of the National Academy of Sciences of the United States of America 93: 10078-10083.
