# Supplementary material for: Modeling T Cell Antigen Discrimination Based on Feedback Control of Digital ERK Responses
Source: PLoS Biol. 2005 Oct 25;3(11):e356. doi: 10.1371/journal.pbio.0030356 (PMC1262625; doi:10.1371/journal.pbio.0030356)
Supplement: Supplementary file 14 [file pbio.0030356.sd005.doc]

**Protocol S5**

In all our computer simulations, we estimated the ppERK response of individual T cells to a given number of ligands. To compare our simulation outputs with the experimental results, one must take into account the noise in the level of presentation of ligands by antigen presenting cells. Indeed, for a given concentration of pulsing peptide, the distribution of ligands being presented by the antigen presenting cell (APC) follows a log-normal distribution with a coefficient of variation of 55% (see Supplementary material S2). Hence, we had to convolve this noise in presentation with the results from our computer simulations to estimate the characteristic time of ERK response of T cells to a typical level of presentation of ligand, as measured experimentally.

We used the following five-term polynomial fit for the computer-simulated dependence of the cell’s response time (*T*) as a function of the number of presented ligands (*x*):

*T(x)=* *318.49-212.13*log(x)+65.166*[log(x)]^2^-9.4663*[log(x)]^3^+0.60105*[log(x)]^4^.*

At a given time *t*, a T cell will respond with ERK phosphorylation if the number *x* of ligands presented by the antigen presenting cell in contact is such that:

*x_0_ (t)< x < x_1_(t),*

where *x_0_ (t)* and *x_1_ (t)* are such that *T (x_0_ (t))= T (x_1_ (t))=t.*

For a typical presentation of *Y* ligands *per* APC, the distribution of ligand presentation follows a log-normal distribution. Thus, we can estimate the probability of a T cell to be activated (noted *q(t,Y*)), by computing the number of APC presenting *x* ligands between *x_0_(t)* and *x_1_(t):*

$$q(t,Y)=\int_{x_{0}(\tau)}^{x_{1}(\tau)} \frac{dx}{x\sigma\sqrt{2}} \exp\left(-\frac{\left(\ln(x)-\ln(Y)\right)^{2}}{2\sigma^{2}}\right),$$

where *σ* is the standard deviation of the distribution of ligand presentation.

For a typical presentation of *Y* ligands *per* APC, the characteristic time *Y* of the ppERK response of T cells is then estimated with:

$$q\left(\tau(Y),Y\right)=\frac{1}{2}\lim_{t\to\infty} q(t,Y).$$

We use a small computer routine to derive this characteristic time *Y* of ppERK response of T cells for different levels *Y* of presentation on APC.

Through this computation taking into account the noise in the level of presentation of the agonist ligands, we explain how the divergence of the response time obtained in our computer simulation (at low ligand number and high ligand number) is abrogated at low levels of presentation and matches well with our experimental results.

Figure: definition of *x_0_ (t)* and *x_1_ (t).* For a given time *t,* only cells that are in contact with APC presenting *x* ligands, such as *x_0_ (t)<x< x_1_ (t),* will have responded. *x_0_ (t)* and *x_1_ (t)* are defined as the points on the simulation curve such as  *T (x_0_ (t))= T (x_1_ (t))=t.*

Figure: noise in the level of presentation of agonist ligand reduces the divergence of the response time at low levels of presentation
